# Familial globular glial tauopathy linked to *MAPT* mutations: molecular neuropathology and seeding capacity of a prototypical mixed neuronal and glial tauopathy

**DOI:** 10.1007/s00401-019-02122-9

**Published:** 2020-01-06

**Authors:** Isidro Ferrer, Pol Andrés-Benito, Maria Victoria Zelaya, Maria Elena Erro Aguirre, Margarita Carmona, Karina Ausín, Mercedes Lachén-Montes, Joaquín Fernández-Irigoyen, Enrique Santamaría, José Antonio del Rio

**Affiliations:** 1 Department of Pathology and Experimental Therapeutics, University of Barcelona, Feixa Llarga sn, 08907 Hospitalet de Llobregat, Spain; 2 Bellvitge University Hospital, Hospitalet de Llobregat, Spain; 3 CIBERNED (Network Centre of Biomedical Research of Neurodegenerative Diseases), Ministry of Economy and Competitiveness, Institute of Health Carlos III, Madrid, Spain; 4 Institute of Neurosciences, University of Barcelona, Barcelona, Spain; 5 Bellvitge Biomedical Research Institute (IDIBELL), Hospitalet de Llobregat, Barcelona, Spain; 6 Pathological Anatomy Department, Hospital of Navarra, Pamplona, Spain; 7 Neurology Department, Complejo Hospitalario de Navarra, Pamplona, Spain; 8 Clinical Neuroproteomics Laboratory, Proteomics Unit, Complejo Hospitalario de Navarra (CHN), Universidad Pública de Navarra (UPNA), IdiSNA, Proteored-ISCIII, NavarrabiomedPamplona, Spain; 9 Molecular and Cellular Neurobiotechnology, Institute of Bioengineering of Catalonia (IBEC), Science Park Barcelona (PCB), Barcelona Institute for Science and Technology, Barcelona, Spain; 10 Department of Cell Biology, Physiology and Immunology, Faculty of Biology, University of Barcelona, Barcelona, Spain

**Keywords:** Globular glial tauopathy, Tau, Astrogliopathy, Oligodendrogliopathy, Phosphoproteome, Seeding and spreading

## Abstract

Globular glial tauopathy (GGT) is a progressive neurodegenerative disease involving the grey matter and white matter (WM) and characterized by neuronal deposition of hyper-phosphorylated, abnormally conformed, truncated, oligomeric 4Rtau in neurons and in glial cells forming typical globular astrocyte and oligodendrocyte inclusions (GAIs and GOIs, respectively) and coiled bodies. Present studies centre on four genetic GGT cases from two unrelated families bearing the P301T mutation in *MAPT* and one case of sporadic GGT (sGGT) and one case of GGT linked to *MAPT* K317M mutation, for comparative purposes. Clinical and neuropathological manifestations and biochemical profiles of phospho-tau are subjected to individual variations in patients carrying the same mutation, even in carriers of the same family, independently of the age of onset, gender, and duration of the disease. Immunohistochemistry, western blotting, transcriptomic, proteomics and phosphoproteomics, and intra-cerebral inoculation of brain homogenates to wild-type (WT) mice were the methods employed. In GGT cases linked to *MAPT* P301T mutation, astrocyte markers *GFAP*, *ALDH1L1*, *YKL40* mRNA and protein, *GJA1* mRNA, and AQ4 protein are significantly increased; glutamate transporter GLT1 (EAAT2) and glucose transporter (SLC2A1) decreased; mitochondrial pyruvate carrier 1 (MPC1) increased, and mitochondrial uncoupling protein 5 (UCP5) almost absent in GAIs in frontal cortex (FC). Expression of oligodendrocyte markers *OLIG1* and *OLIG2mRNA,* and myelin-related genes *MBP*, *PLP1*, *CNP*, *MAG*, *MAL*, *MOG,* and *MOBP* are significantly decreased in WM; CNPase, PLP1, and MBP antibodies reveal reduction and disruption of myelinated fibres; and SMI31 antibodies mark axonal damage in the WM. Altered expression of AQ4, GLUC-t, and GLT-1 is also observed in sGGT and in GGT linked to *MAPT* K317M mutation. These alterations point to primary astrogliopathy and oligodendrogliopathy in GGT. In addition, GGT linked to *MAPT* P301T mutation proteotypes unveil a proteostatic imbalance due to widespread (phospho)proteomic dearrangement in the FC and WM, triggering a disruption of neuron projection morphogenesis and synaptic transmission. Identification of hyper-phosphorylation of variegated proteins calls into question the concept of phospho-tau-only alteration in the pathogenesis of GGT. Finally, unilateral inoculation of sarkosyl-insoluble fractions of GGT homogenates from GGT linked to *MAPT* P301T, sGGT, and GGT linked to *MAPT* K317M mutation in the hippocampus, corpus callosum, or caudate/putamen in wild-type mice produces seeding, and time- and region-dependent spreading of phosphorylated, non-oligomeric, and non-truncated 4Rtau and 3Rtau, without GAIs and GOIs but only of coiled bodies. These experiments prove that host tau strains are important in the modulation of cellular vulnerability and phenotypes of phospho-tau aggregates.

**Electronic supplementary material:**

The online version of this article (10.1007/s00401-019-02122-9) contains supplementary material, which is available to authorized users.

## Introduction

Globular glial tauopathy (GGT) identifies a group of neurodegenerative diseases with abnormal accumulation of phospho-tau in neurons and phospho-tau-containing globular glial inclusions (GGIs) in astrocytes and oligodendrocytes [1, 3, 7, 45, 63, 70]. GGIs in astrocytes (GAIs) differ from tufted astrocytes, astrocytic plaques, thorn-shaped astrocytes, and fibrillar astrocytes containing phospho-tau in other tauopathies; however, tufted astrocytes and, rarely, astrocytic plaques can be found in GGTs. Globular inclusions in oligodendrocytes (GOIs) differ from coiled bodies, although both types of oligodendroglial inclusions may co-exist in GGT. Western blotting of sarkosyl-insoluble fractions reveals a typical 4Rtau band pattern consisting of two bands of 68 kDa and 64 kDa and several lower bands of about 35 kDa of phosphorylated tau [3, 7, 38, 45].

The majority of GGTs are sporadic [1], but a few familial cases have been reported linked to different mutations in the microtubule-associated protein tau gene (*MAPT*): N296H in exon 10 [62], R5H in exon 1 [56], K317M in exon 11 [40, 135], K317N in exon 11 [111], P301L in exon 10 [10, 44, 112], and IVS10 + 16 [44]. Recently, we have reported five new cases of familial GGT from two unrelated pedigrees bearing the P301T mutation in *MAPT* [28]. A typical 4Rtau band pattern consisting of two bands of 68 kDa and 64 kDa, and several lower bands of about 35 kDa, is also found in familial GGTs [28, 111, 135]. GGT has been classified according to three subtypes [1]. Type I has predominant frontal symptoms and pathology, abundant GOIs, and scarce coiled bodies and GAIs; type III is manifested by severe involvement of the frontal cortex, motor cortex, temporal cortex, and anterior horn of the spinal cord, and presents abundant GAIs in the cerebral cortex, and GOIs and colied bodies in the white matter; and type II has severe involvement of the motor cortex and pyramidal tracts, abundant GOIs and coiled bodies in the white matter but low numbers of GAIs in the cerebral cortex [1]. Involvement of the frontal white matter is usually severe in types I and III, and involvement of the substantia nigra occurs in types II and III [3].

Beyond the clinical manifestations and typical neuropathological hallmarks, little is known about molecular alterations in GGT and possible implications in the pathogenesis of the disease. Moreover, although GAIs and GOIs are typical of GGT, nothing is known about the functional alterations linked to the pathology of astrocytes and oligodendrocytes. Recent studies have shown the seeding capacity of GGT homogenates in vivo and in vitro [13, 36, 41]. However, little attention has been paid to the characteristics of tau deposits linked to the host tau.

Our hypotheses are that (a) astrocytes and oligodendrocytes are principal targets and dysfunctional players in the pathogenesis of GGT; (b) GGT, like other neurodegenerative diseases with hyper-phosphorylated tau deposition, is a more generalized disease with hyper-phosphorylation of a large number of proteins in addition to tau; and (c) inoculation in wild-type (WT) mice of abnormal mutant tau from human GGT brain homogenates has the capacity to recruit murine tau to generate seeding and spreading largely dependent on the characteristics of host tau.

Therefore, the present study is geared to learning about (i) characterization of phospho-tau species in GGT; (ii) molecular alterations in astrocytes and oligodendrocytes, (iii) proteostatic dearrangements in addition to tau in the brain of GGT cases, and (iv) capacities of seeding and spreading for abnormal tau from GGT homogenates containing human mutant tau after inoculation in the brain of WT mice, and characterization of inclusions in glial cells.

For these purposes, we focused the study on recently reported genetic GGT cases from two unrelated pedigrees bearing the P301T mutation in *MAPT* [28]. For comparative purposes, we also analysed one sporadic GGT presenting with primary progressive aphasia [case 1, 38], and another genetic GGT case manifested with frontotemporal dementia, parkinsonism, and motor neuron disease linked to K317M mutation in *MAPT* [135].

## Materials and methods

### Familial GGT linked to P301T mutation in *MAPT*

Patients belonged to two apparently unrelated pedigrees from a small region of the north of Spain. Case 1 belongs to pedigree 1; and cases 2, 3 and 4, two brothers and one sister from seven siblings, to pedigree 2. Patients bore the same P301T mutation in the *MAPT* gene; detailed clinical manifestations are reported in the original description [28].

#### Case 1

The patient was a man aged 45 years with progressive gait disturbance. The neurological examination showed pyramidal syndrome, and a diagnosis of primary lateral sclerosis was initially established. He developed cognitive decline, with non-fluent speech, paresis of vertical and horizontal gaze movements, axial rigidity, and asymmetric tetraparesis with severe spasticity and dystonic postures. He died at the age of 49 with a clinical diagnosis of progressive supranuclear palsy plus primary lateral sclerosis. He was an only child; his mother died at the age of 70 years with a clinical diagnosis of probable corticobasal degeneration. His grandfather had suffered from dementia and died at the age of 53.

#### Case 2

The patient was a 49-year-old man who had suffered from gait instability, loss of motor coordination of the right hand, alien hand, myoclonus, and dystonic movements in the right extremities. This was followed by cognitive impairment and frontotemporal dementia. The clinical diagnosis was corticobasal syndrome. He died at the age of 55.

#### Case 3

The patient was a 43-year-old man with a short history of speech difficulties, rigidity, and bradikynesia in the right arm and leg, and alien hand, which were accompanied by aggressive behaviour and cognitive decline. The clinical diagnosis was corticobasal syndrome. He died at the age of 47.

#### Case 4

The patient was a woman aged 55 who suffered from progressive apathy, anhedonia, and mood disorder progressing to stereotypic motor behaviour and dementia. The clinical diagnosis was frontotemporal dementia. She died at the age of 61.

The mother, one aunt, and two uncles of seven siblings, as well as the grandmother, had suffered from a neurological disease with variable clinical symptoms and predominant cognitive decline.

### Other GGT cases: sporadic GGT and familial GGT linked to *MAPT* K317M mutation

#### Sporadic GGT (sGGT)

The patient was a 66-year-old right-handed woman with primary progressive aphasia. The cerebral magnetic resonance imaging (MRI) demonstrated cortical atrophy of the right perisylvian region and the left temporal lobe, together with hyperintense periventricular signals in the white matter. This was followed by buco-lingual apraxia, spasmodic laughter, difficulty in swallowing, and deterioration of cognitive functions. The patient progressively lost mobility and suffered from cognitive deterioration, until death at the age of 81, 12 years after the beginning of aphasia. Additional clinical details can be found in [38], case 1.

#### Familial GGT linked to *MAPT* K317M mutation

The patient was a 57-year-old woman suffering from dysarthria, bradykinesia, parkinsonism, pyramidal syndrome, slowing of ocular saccades, ideomotor apraxia, mutism, echolalia, and mirror movements, who died 11 years after the initiation of symptoms. One brother was affected by the same disease. The genetic study of a post-mortem sample of the cerebral cortex identified a K317M mutation in *MAPT*. Further details are described in [135], case I/III-21.

### Control cases

Unrelated controls with no neurological symptoms and no brain lesions in the post-mortem neuropathological study (7 men and 3 women; mean age: 67 ± 7 years) were processed and assessed in parallel.

The frontal cortex of one patient with Alzheimer’s disease (one man aged 78 years) Braak and Braak stage VI of neurofibrillary tangle degeneration was used for comparison in western blotting studies.

### Neuropathological study

Post-mortem human brains were obtained following the guidelines of the Spanish legislation on this matter (Real Decreto 2011/1716) and approval of the local ethics committees.

Immediately after removal of the brain from the skull, fresh samples from the frontal cortex and frontal white matter were frozen and stored at − 80 °C for biochemical studies. The rest of the brain was fixed in 4% buffered formalin, and selected samples of the brain and spinal cord were embedded in paraffin. Tissue sections, 4 μm thick, were obtained with a sliding microtome. The sections were stained with haematoxylin and eosin, Klüver–Barrera, and Sudan black, or processed for immunohistochemistry. Additional tissue samples of the frontal cortex and underlying white matter were processed for Gallyas staining.

#### Immunohistochemistry

The sections were boiled in citrate buffer (20 min) to retrieve protein antigenicity. Endogenous peroxidases were blocked by incubation in 10% methanol–1% H_2_O_2_ solution (15 min) followed by 3% normal horse serum solution. Then, the sections were incubated at 4 °C overnight with one of the primary antibodies listed in Table 1. Following incubation with the primary antibody, the sections were incubated with EnVision + system peroxidase (Dako, Agilent Technologies, Santa Clara, CA, USA) for 30 min at room temperature. The peroxidase reaction was visualized with diaminobenzidine and H_2_O_2_. Control of the immunostaining included omission of the primary antibody; no signal was obtained following incubation with only the secondary antibody.

**Table 1:** Characteristics of the antibodies used for immunohistochemistry and double-labelling immunofluorescence in human and mouse brain samples

Antibody	Mono-/polyclonal	Dilution	Supplier	Country
4Rtau	Monoclonal	1:50	Merck-Millipore	Billerica, MA, USA
3Rtau	Monoclonal	1:800	Merck-Millipore	Billerica, MA, USA
P-tauThr181	Rabbit polyclonal	1:50	Cell Signaling	Danvers, MA, USA
P-tauSer422	Rabbit polyclonal	1:1000	Thermo Fisher	Waltham, MA, USA
AT8 (Ser202/Thr205)	Monoclonal	1:50	Innogenetics	Ghent, BE
MC-1 (aa312-322)	Monoclonal	1:50	Dr. Peter Davies	USA
Tau-C3 (tr Asp421)	Monoclonal	1:300	Abcam	Cambridge, UK
tau-22 (oligomeric)	Rabbit polyclonal	1:200	Merck-Millipore	Billerica, MA, USA
NF-RT97 (NF 200 kDa)	Monoclonal	1:50	Novocastra	Newcastle, UK
SMI31 (phosphorylated neurofilament H)	Monoclonal	1:300	Biolegend	San Diego, CA, USA
NeuN	Monoclonal	1:100	Merck-Millipore	Billerica, MA, USA
GFAP: glial fibrillary acidic protein	Rabbit polyclonal	1:500	Dako	Glostrup, DK
Olig2	Rabbit polyclonal	1:500	Abcam	Cambridge, UK
NG2	Rabbit polyclonal	1:200	Sigma-Aldrich, Merck	Darmstadt, GE
Iba1	Rabbit polyclonal	1:1000	Wako	Richmond, VA, USA
αB-crystallin	Monoclonal	1:500	Novocastra-Leica	Barcelona, Spain
YKL40	Rabbit polyclonal	1:200	Invitrogen	Carlsbad, CA, USA
AQ4	Monoclonal	1:400	Sigma	St Louis, Missouri, USA
GLT1 (glutamate transporter) EAAT2	Guinea pig	1:100	Merck-Millipore	Billerica, MA, USA
MPC1: mitochondrial pyruvate carrier 1	Rabbit polyclonal	1:100	Cell Signaling	Danvers, MA, USA
UCP4: mitochondrial uncoupling protein 4	Rabbit polyclonal	1:100	Abyntek	Derio, BI, Spain
UCP5: mitochondrial uncoupling protein 5	Rabbit polyclonal	1:25	Novus Biologicals/Bionova	Madrid, Spain
GLUC-t (glucose transporter) SLC2A1	Rabbit polyclonal	1:100	Abcam	Cambridge, UK
CNPase	Monoclonal	1:100	Sigma-Aldrich, Merck	Darmstadt, GE
Myelin basic protein (MBP)	Monoclonal	1:1000	Abcam	Cambridge, UK
Proteolipid protein (PLP1)	Monoclonal	1:100	LifeSpan Biosciences	Seattle, WA, USA
Histone H3 (di-methyl K9) H3K9me2	Monoclonal	1:50	Abcam	Cambridge, UK
Histone H4 (acetyl K12) H4K12ac	Rabbit polyclonal	1:500	Abcam	Cambridge, UK
p38-P Thr180-Tyr182	Rabbit polyclonal	1:100	Cell Signaling	Danvers, MA, USA
β-Amyloid	Monoclonal	1:50	Dako	Glostrup, DK
α-Synuclein	Rabbit polyclonal	1:500	Chemicon, Merck-Millipore	Billerica, MA, USA
TDP-43	Rabbit polyclonal	1:200	Abcam	Cambridge, UK
Ubiquitin	Rabbit polyclonal	1:200	Dako	Glostrup, DK
p62	Guinea pig polyclonal	1:100	Progen, RA Biopharm	Darmstadt, GE

Quantification of blood vessels in sections processed for GLUC-t (glucose transporter) immunohistochemistry was carried out as follows: images were acquired at a magnification 200× in five different areas of the frontal cortex in every case. The number of capillaries was expressed as the mean values ± SEM in area of 0.15 mm^2^. Quantification of GLUC-t-positive capillaries was performed using Fiji ImageJ software. Statistical analysis was performed using GraphPad Prismv5 software. Results were analysed using the Student’s *t* test.

#### Double-labelling immunofluorescence and confocal microscopy

De-waxed sections, 4 μm thick, were stained with a saturated solution of Sudan black B (Merck, Glostrup, DE) for 15 min to block the autofluorescence of lipofuscin granules present in cell bodies and then rinsed in 70% ethanol and washed in distilled water. The sections were incubated at 4 °C overnight with combinations of primary antibodies AT8 or P-tauThr181, and GFAP, YKL40, αB-crystallin, Tau-C3, MPC1 (mitochondrial pyruvate carrier 1), UCP4 (mitochondrial uncoupling protein 4), UCP5 (mitochondrial uncoupling protein 4), histone H4 (di-methyl K9) and histone H3 (acetyl K12), and phosphorylate kinase p38 (p38-P Thr180-Tyr182). The characteristics of the antibodies are listed in Table 1. After washing, the sections were incubated with Alexa488 or Alexa546 (1:400, Molecular Probes, Eugene, OR, USA) fluorescence secondary antibodies against the corresponding host species. Nuclei were stained with DRAQ5™ (dilution 1:2000, BioStatus, Loughborough, UK). After washing, the sections were mounted in Immuno-Fluore mounting medium (ICN Biomedicals, Irvine, CA, USA), sealed, and dried overnight. Sections were examined with a Leica TCS-SL confocal microscope [40].

Co-localization of 2 proteins labelled with specific antibodies and examined with the confocal microscope was assessed by counting cells expressing both antigens in relation to the number of cells stained with each one of the antibodies in 5 selected fields per section at a magnification of 600 in every case. Quantitative studies were restricted to frontal cortex area 8 and underlying white matter. Values were expressed as the percentage in reference to the more abundant protein because the less abundant protein represented a subset of the former (e.g. percentage of P-tauThr181-positive astrocytes containing tau-C3) [40].

#### Western blotting of sarkosyl-insoluble fractions

Frozen samples of about 1 g from frontal cortex in every case, and frontal cortex and subcortical white matter separately from case 1, were lysed in 10 volumes (w/v) with cold suspension buffer (10 mM Tris–HCl, pH 7.4, 0.8 M NaCl, 1 mM EGTA) supplemented with 10% sucrose, protease, and phosphatase inhibitors (Roche, GE). The homogenates were first centrifuged at 20,000×*g* for 20 min (Ultracentrifuge Beckman with 70Ti rotor), and the supernatant (S1) was saved. The pellet was re-homogenized in 5 volumes of homogenization buffer and re-centrifuged at 20,000×*g* for 20 min (Ultracentrifuge Beckman with 70Ti rotor). The two supernatants (S1 + S2) were then mixed and incubated with 0.1% *N*-lauroylsarkosynate (sarkosyl) for 1 h at room temperature while being shaken. Samples were then centrifuged at 100,000×*g* for 1 h (Ultracentrifuge Beckman with 70Ti rotor). Sarkosyl-insoluble pellets (P3) were re-suspended (0.2 ml/g) in 50 mM Tris–HCl (pH 7.4). Protein concentrations were quantified with the bicinchoninic acid assay (BCA) assay (Pierce, Waltham, MA, USA). Samples were mixed with loading sample buffer and heated at 95 °C for 5 min. Sixty micrograms of protein was separated by electrophoresis in SDS-PAGE gels and transferred to nitrocellulose membranes (200 mA per membrane, 90 min). The membranes were blocked for 1 h at room temperature with 5% non-fat milk in TBS containing 0.2% Tween and were then incubated with one of the primary antibodies: anti-tau Ser422 (diluted 1:1000), anti-4Rtau (diluted 1:1000), and anti-3Rtau (diluted 1:1000). After washing with TBS-T, blots were incubated with the appropriate secondary antibody (anti-mouse/anti-rabbit IgG conjugated with horseradish peroxidase, diluted at 1:2000, DAKO, DE) for 45 min at room temperature. Immune complexes were revealed by incubating the membranes with chemiluminescence reagent (Amersham, GE Healthcare, Buckinghamshire, UK) [37]. Samples of the frontal cortex from one case with AD stage VI of Braak and Braak and one age-matched control without tau pathology were processed in the same way for comparative purposes.

Sarkosyl-insoluble fractions of the frontal cortex and subcortical white matter, and soluble fractions of the frontal cortex, were also used for inoculation in mice.

#### RNA purification, retro-transcription reaction, and RT-qPCR

RNA from frontal cortex and the white matter from the four familiar GGT cases linked to the *MAPT* P301T mutation was extracted following the instructions of the supplier (RNeasy Mini Kit, Qiagen^®^ GmbH, Hilden, Germany). Age-matched control cases (*n* = 10) without neurological or neuropathological lesions were processed in parallel. RNA integrity and 28S/18S ratios were determined with the Agilent Bioanalyzer (Agilent Technologies Inc, Santa Clara, CA, USA) to assess RNA quality, and the RNA concentration was evaluated using a NanoDrop™ Spectrophotometer (Thermo Fisher Scientific, Waltham, MA, USA). RIN values of GGT cases and controls were between 5.1 and 7.3. Complementary DNA (cDNA) preparation used the High-Capacity cDNA Reverse Transcription kit (Applied Biosystems, Foster City, CA, USA) following the protocol provided by the supplier. Parallel reactions for each RNA sample were run in the absence of MultiScribe Reverse Transcriptase to assess the lack of contamination of genomic DNA. TaqMan RT-qPCR assays were performed in duplicate for each gene on cDNA samples in 384-well optical plates using an ABI Prism 7900 Sequence Detection system (Applied Biosystems, Life Technologies, Waltham, MA, USA). For each 10 μL TaqMan reaction, 4.5 μL cDNA was mixed with 0.5 μL 20× TaqMan Gene Expression Assays and 5 μL of 2× TaqMan Universal PCR Master Mix (Applied Biosystems). Values of β-glucuronidase (*GUS-β*) were used as internal controls for normalization. TaqMan probes and references are listed in Table 2. The parameters of the reactions were 50 °C for 2 min, 95 °C for 10 min, and 40 cycles of 95 °C for 15 s and 60 °C for 1 min. Finally, capture of all TaqMan PCR data used the Sequence Detection Software (SDS version 2.2.2, Applied Biosystems). For the data analysis, threshold cycle (CT) values for each sample were processed to obtain the double delta CT (ΔΔCT) values. First, delta CT (ΔCT) values were calculated as the normalized CT values of each target gene in relation to the CT of endogenous controls *GUS-β*. Then, ΔΔCT values were obtained from the ΔCT of each sample minus the mean ΔCT of the population of control samples. Results were analysed using Student's *t* test [4].

**Table 2:** Gene symbols and TaqMan probes used in frontal cortex and subcortical white matter

Gene	Full name	Reference
*ALDH1L1*	Aldehyde dehydrogenase 1 family member L1	Hs01003842_m1
*AQP4*	Aquaporin-4	Hs00242342_m1
*CNP*	2′,3′-Cyclic nucleotide 3′ phosphodiesterase	Hs00263981_m1
*GFAP*	Glial fibrillary acidic protein	Hs00909233_m1
*GJA1*	Gap junction alpha-1 protein/connexin-43	Hs00748445_s1
*GUS-β*	β-Glucuronidase	Hs00939627_m1
*MAG*	Myelin-associated glycoprotein	Hs01114387_m1
*MAL*	Mal, T-cell differentiation protein	Hs00360838_m1
*MBP*	Myelin basic protein	Hs00921945_m1
*MCT1*	Solute carrier family 16 (monocarboxylic acid transporters), member 1	Hs01560299_m1
*MOBP*	Myelin-associated oligodendrocyte basic protein	Hs01094434_m1
*MOG*	Myelin oligodendrocyte glycoprotein	Hs01555268_m1
*MPC1*	Mitochondrial pyruvate carrier 1	Hs00211484_m1
*MYRF*	Myelin regulatory factor	Hs00973739_m1
*NG2*	Neural/glial antigen 2	Hs00426981_m1
*OLIG1*	Oligodendrocyte transcription factor 1	Hs00744293_s1
*OLIG2*	Oligodendrocyte lineage transcription factor 2	Hs00377820_m1
*PLP1*	Proteolipid protein 1	Hs00166914_m1
*SLC1A2*	Solute carrier family 1 (glial high affinity glutamate transporter), member 2	Hs01102423_m1
*SLC2A1*	Solute carrier family 2 (facilitated glucose transporter), member 1	Hs01102423_m1
*SOX-10*	SRY-Box 10	Hs00366918_m1
*UCP4*	Mitochondrial uncoupling protein 4	Hs00188687_m1
*UCP5*	Mitochondrial uncoupling protein 5	Hs00605850_m1
*YKL40*	Chitinase 3 like 1	Hs01072228_m1

### Neuroanatomical proteomics

Frontal cortex and underlying white matter specimens derived from the same control and GGT cases linked to *MAPT* P301T mutation were homogenized separately in lysis buffer containing 7 M urea, 2 M thiourea, 4% (w/v), and 50 mM DTT supplemented with protease and phosphatase inhibitors. The homogenates were spun down at 100,000×*g* for 1 h at 15 °C. After protein precipitation, protein concentration in the supernatants was measured with the Bradford assay kit (Biorad).

#### Protein digestion and peptide iTRAQ labelling

An iTRAQ (isobaric Tags for Relative and Absolute Quantitation)-based quantitative proteomic analysis was performed for frontal cortex and white matter as previously described [72, 136]. iTRAQ labelling of each sample was performed according to the manufacturer’s protocol (Sciex). Briefly, equal amounts of protein (100 μg) from each sample were reduced with 50 mM tris (2-carboxyethyl) phosphine (TCEP) at 60 °C for 1 h. Cysteine residues were alkylated with 200 mM methyl methanethiosulfonate (MMTS) at room temperature for 15 min. Protein enzymatic cleavage was carried out with trypsin (Promega; 1:20, w/w) at 37 °C for 16 h. For both iTRAQ experiments, each tryptic digest was labelled with one isobaric amine-reactive tag as follows: Tag113, control-1; Tag114, control-2; Tag115, control-3; Tag116, control-4; Tag117, GGT-1; Tag118, GGT-2; Tag119, GGT-3; Tag121, GGT-4. After 2 h incubation, the sets of frontal cortex- and white matter-labelled samples were independently pooled and evaporated in a vacuum centrifuge.

#### Phosphopeptide enrichment and LC–MS/MS

The enrichment of phosphorylated peptides was performed applying the SIMAC protocol as previously described [119]. Unbound peptide pools (non-modified peptides) were dried in a vacuum centrifuge and reconstituted with 40 μL of 5 mM ammonium bicarbonate (ABC), pH 9.8, and injected into an ÄKTA pure 25 system (GE Healthcare Life Sciences) with a high pH stable X-Terra RP18 column (C18; 2.1 mm × 150 mm; 3.5 μm) (Waters). Mobile phases were 5 mM ammonium formate in 90% ACN at pH 9.8 (buffer B) and 5 mM ammonium formate in water at pH 9.8 (buffer A). Column gradient was developed in an 80-min three-step gradient from 5% B to 30% B in 5 min, 30% B to 60% B in 40 min, 15 min in 60% B, and 60% B to 90% B in 20 min. Column was equilibrated in 95% B for 30 min and 2% B for 10 min. Thirteen fractions were collected and evaporated under vacuum. Peptide fractions were reconstituted into a final concentration of 0.5 µg/µL of 2% ACN, 0.5% FA, 97.5% MilliQ-water prior to mass spectrometric analysis. Then, peptide mixtures were separated by reverse phase chromatography using an Eksigent nanoLC ultra 2D pump fitted with a 75-μm ID column (Eksigent 0.075 × 250). Samples were first loaded for desalting and concentration into a 2-cm-length 100-μm ID precolumn packed with the same chemistry as the separating column. Mobile phases were 100% water, 0.1% formic acid (FA) (buffer A), and 100% acetonitrile 0.1% FA (buffer B). Non-modified peptide fractions were analysed under the following conditions. Column gradient was developed in a 135-min three-step gradient from 2% B to 30% B in 90 min, from 30% B to 40% B in 10 min, and from 40 to 80% in 10 min. Column was equilibrated in 97% B for 3 min and 2% B for 23 min. During all the process, precolumn was in line with column and flow maintained all along the gradient at 300 nl/min. Eluting peptides from the column were analysed using a 5600 Triple-TOF system (Sciex). Data acquisition was carried out using a survey scan in a mass range from 350 *m*/*z* to 1250 *m*/*z* for 250ms. The top 35 peaks were selected for fragmentation. Minimum accumulation time for MS/MS was set at 11 ms giving a total cycle time of 3.8 s. Product ions were scanned in a mass range from 100 *m*/*z* up to 1500 *m*/*z* and excluded for further fragmentation for 15 s. In the case of fractions that contained the phosphorylated peptides derived from FC and WM, column gradient was developed in 140-min two-step gradient from 2 to 35% B in 100 min and from 35 to 70% in 20 min. Column was equilibrated in 95% B for 5 min and 2% B for 15 min. Precolumn was in line with column and flow maintained all along the gradient at 300 nl/min. Eluting peptides from the column were analysed using a 5600 Triple-TOF system (Sciex) following the same conditions as the non-modified peptides.

#### Data analysis

The raw MS/MS spectra searches were processed using the MaxQuant software (v.1.5.8.3) [120] and searched against the Uniprot proteome reference for *Homosapiens* (Proteome ID: UP000005640_9606, February 2019). The parameters used were as follows: initial maximum precursor (25 ppm) fragment mass deviations (40 ppm); variable modification (methionine oxidation and N-terminal acetylation) and fixed modification (MMTS); enzyme (trypsin) with a maximum of 1 missed cleavage; minimum peptide length (7 amino acids); and false discovery rate (FDR) for PSM and protein identification (1%). Frequently observed laboratory contaminants were removed. Protein identification was considered valid with at least one unique or “razor” peptide. The protein quantification was calculated using at least 2 razor + unique peptides, and statistical significance was calculated with a two-way Student *t* test (*p* < 0.05). A 1.3-fold change cut-off was used. Proteins with iTRAQ ratios below the low range (0.77) were considered to be down-regulated, whereas those above the high range (1.3) were considered to be up-regulated. Additionally, in the analysis of the frontal cortex and white matter phosphorylated fractions, phosphoserine, phosphothreonine, and phosphotyrosine were chosen as variable modifications for database searching. The Perseus software (version 1.5.6.0) [120] was used for statistical analysis and data visualization. Search result files and MS raw data were deposited in the ProteomeXchange Consortium (https://proteomecentral.proteomexchange.org) via the PRIDE partner repository with the dataset identifiers PXD015768 (Reviewer account details: Username: reviewer51422@ebi.ac.uk; Password: vhhPC599).

The identification of significantly dysregulated regulatory/metabolic pathways in frontal cortex and white matter proteomic datasets was performed using Metascape [138]. The interactome of tau was analysed using FpClass (https://dcv.uhnres.utoronto.ca/FPCLASS/) [66], a database of predicted protein–protein interactions (PPIs), the curated Biological General Repository for Interaction Datasets (BioGRID: https://thebiogrid.org) [95], and STRING [110].

### Animals and tissue processing

Wild-type C57BL/6 mice from our colony were used. All animal procedures were carried out following the guidelines of the European Communities Council Directive 2010/63/EU and with the approval of the ethical committee of the University of Barcelona, Spain.

Four series of mice were unilaterally inoculated with sarkosyl-insoluble fractions from the frontal cortex of GGT linked to *MAPT* P301T mutation (case 1): (i) mice aged 12 months in the right hippocampus and killed at 18–19 months (survival 6–7 months), *n* = 4; (ii) mice aged 7 months in the right corpus callosum and killed at the age of 11 months (survival 4 months), *n* = 4; (iii) mice aged 12 months in the right corpus callosum and killed at the age of 18 months (survival 6 months), *n* = 4; and mice aged 10 months in the right caudate/putamen (CPu) and killed 5 months later, *n* = 4. Another series was inoculated in the right hippocampus with sarkosyl-insoluble fractions from the white matter of GGT case 1 at the age of 7 months and killed at the age of 14 months (survival 7 months), *n* = 4. Control mice included two mice inoculated in the right hippocampus with sarkosyl-soluble fractions and one mouse inoculated with 50 mM Tris–HCl (pH 7.4) as vehicle at the age of 7 months and killed four months later.

For comparative purposes, WT mice aged 3–4 months were unilaterally inoculated in the right hippocampus or the corpus callosum with sarkosyl-insoluble fractions of frontal cortex homogenates from sGGT (mice, *n* = 3), and frontal cortex from GGT linked to *MAPT* K317M mutation (mice, *n* = 2), and killed at the age of 18–19 months (survival 6–7 months).

The total number of animals, males, and females was 24.

#### Inoculation into the hippocampus, lateral corpus callosum, and caudate/putamen

The reasons for inoculating in these particular regions were to assess: (a) neuronal transmission following unilateral injection in the hippocampus, (b) transmission along white matter tracts and involvement of the white matter following unilateral inoculation in the corpus callosum, and (c) regional vulnerability comparing inoculation in the hippocampus and the caudate/putamen.

Mice were deeply anesthetized by intra-peritoneal ketamin/xylazine/buprenorphine cocktail injection and placed in a stereotaxic frame after assuring lack of reflexes. Intra-cerebral injections were done using a Hamilton syringe; the coordinates for hippocampal injections were − 1.9 AP; −/+ 1.4 ML relative to Bregma and − 1.5 DV from the dural surface; the coordinates for lateral corpus callosum inoculations were − 1.9 AP; −/+ 1.4 ML relative to Bregma and − 1.0 DV from the dural surface; the coordinates for caudate/putamen (CPu) were 0.14 AP; − /+ 2 relative to Bregma and − 2.5 from the dural surface [96]. A volume of 1.5 µL was injected at a rate of 0.05 µL/min in the hippocampus and CPu, and 1.2 µL was injected at a rate of 0.1 µL/min in the corpus callosum. The syringe was retired slowly over a period of 10 min to avoid leakage of the inoculum. Following surgery, the animals were kept in a warm blanket and monitored until they recovered from the anaesthesia. Carprofen analgesia was administered immediately after surgery and once a day during the following two days. Animals were housed individually with full access to food and water.

#### Tissue processing

Animals were killed under anaesthesia, and the brains were rapidly fixed with 4% paraformaldehyde in phosphate buffer and embedded in paraffin. Consecutive serial coronal sections 4 μm thick of the whole brain were obtained with a sliding microtome. De-waxed sections were stained with haematoxylin and eosin, stained with Gallyas silver method, or processed for immunohistochemistry using the antibodies AT8, anti-4Rtau, anti-3Rtau, MC-1, Tau-C3, oligomeric tau-22, PLP1 and SMI31, in addition to GFAP for reactive astrocytes and Iba1 for microglia. Following incubation with the primary antibody, the sections were incubated with EnVision + system peroxidase for 30 min at room temperature. The peroxidase reaction was visualized with diaminobenzidine and H_2_O_2_. Control of the immunostaining included omission of the primary antibody; no signal was obtained following incubation with only the secondary antibody. The specificity of 3Rtau and 4Rtau antibodies in mice was tested in coronal sections of the brain, cerebellum, and brainstem of P301S transgenic mice aged 8–9 months [84]. Tau-immunoreactive inclusions in mice expressing mutant human 4Rtau were positive with anti-4Rtau antibodies but negative with anti-3Rtau antibodies.

Double-labelling immunofluorescence was carried out on de-waxed sections, 4 μm thick, which were stained with a saturated solution of Sudan black B (Merck, DE) for 15 min to block autofluorescence of lipofuscin granules present in cell bodies, and then rinsed in 70% ethanol and washed in distilled water. The sections were boiled in citrate buffer to enhance antigenicity and blocked for 30 min at room temperature with 10% foetal bovine serum diluted in PBS. Then, the sections were incubated at 4 °C overnight with combinations of AT8 and one of the following primary antibodies: GFAP, Iba1, Olig2, phosphorylated p38 at Thr180-Tyr182 (p38-P Thr180-Tyr182), and anti-histone H4 (acetyl K12). Other sections were immunostained with anti-phospho-tauThr181, anti-NeuN, and anti-histone H3 (di-methyl K9) (see Table 1 for the characteristics of the antibodies). After washing, the sections were incubated with Alexa488 or Alexa546 fluorescence secondary antibodies against the corresponding host species. Nuclei were stained with DRAQ5™. Then, the sections were mounted in Immuno-Fluore™ mounting medium, sealed, and dried overnight. Sections were examined with a Leica TCS-SL confocal microscope. Quantitative studies were carried in the ipsilateral corpus callosum in three non-consecutive sections per case using double-labelling immunofluorescence and confocal microscopy. Data were expressed as the percentage of oligodendrocytes (as revealed with the Olig2 antibody) with tau deposits (as seen with the antibody AT8) compared with the total number of oligodendrocytes in the same field following the same protocol as used in human brains.

In situ end-labelling of nuclear DNA fragmentation (ApoptTag^®^ peroxidase in situ apoptosis detection kit, Merck) was used to visualize apoptotic cells. The brains of newborn irradiated rats (2Gys) with a survival time of 24 h were fixed in 4% paraformaldehyde and embedded in paraffin; de-waxed sections were processed in parallel with tissue samples from inoculated mice and used as positive controls of apoptosis.

## Results

### Neuropathological characteristics of GGT linked to *MAPT* P301T mutation

Frontal atrophy was found in every case, accompanied by parietal atrophy in cases of the second pedigree. The temporal cortex and hippocampus were atrophic in case 1. Microscopic examination revealed common alterations but with variable distribution and intensity. Neuron loss, spongiosis in the upper cortical layers, occasional ballooned neurons, marked astrocytic gliosis (revealed with anti-GFAP antibodies), and mild microgliosis (revealed with Iba1 antibody) were severe in the cerebral cortex; neuron loss also occurred in diencephalic nuclei and nuclei of the basal forebrain, and in the substantia nigra and locus ceruleus with neuromelanin granules in the neuropil. αB-crystallin immunoreactivity was seen in ballooned neurons and in a subpopulation of reactive astrocytes. Myelin pallor, as seen with Klüver–Barrera staining, and PLP1, MBP and CNPase immunohistochemistry, together with loss of neurofilaments (as seen with RT97 immunohistochemistry), was found in the white matter, corpus callosum, internal capsule, and pyramidal tracts. Sudan black identified occasional perivascular macrophages around blood vessels and small granules in the lobar white matter, internal capsule, and corpus callosum.

Phospho-tau deposits in neurons, astrocytes, oligodendrocytes, and threads were present in every case, although with variable distribution and morphology from one case to another. Most dramatic deposits occurred in case 1.

Neurons with phospho-tau deposits were localized in the prefrontal cortex, motor cortex, primary sensory cortex, parietal cortex, temporal cortex, insula, occipital cortex, hippocampus (including CA1, CA2, CA3 regions and hilus), dentate gyrus, subiculum, entorhinal cortex, Meynert nucleus, caudate, putamen, pallidum, thalamus, substantia nigra, midbrain tectum, locus ceruleus, pontine nuclei, pontine tectum, and anterior horn of the spinal cord.

Globular astrocytic inclusions (GAIs) were very abundant in the same regions as described for phospho-tau deposits in neurons in case 1. GAIs were more rarely seen in the anterior horn of the spinal cord. GAIs were much lower in numbers in the cerebral cortex and absent in the other brain regions in cases 2, 3, and 4.

Globular oligodendroglial inclusions (GOIs) and coiled bodies were abundant in most grey matter regions, and in the subcortical white matter, corpus callosum, internal capsule, and fascicles of the basal forebrain in case 1. GOIs and coiled bodies were less common or absent in the white matter and other brain regions in the three cases of pedigree 2.

Phospho-tau-immunoreactive threads in the cerebral cortex and white matter paralleled the distribution and intensity of grey and white lesions in every individual case; most severe lesions were seen in case 1 when compared with cases of pedigree 2.

**Fig. 1:**
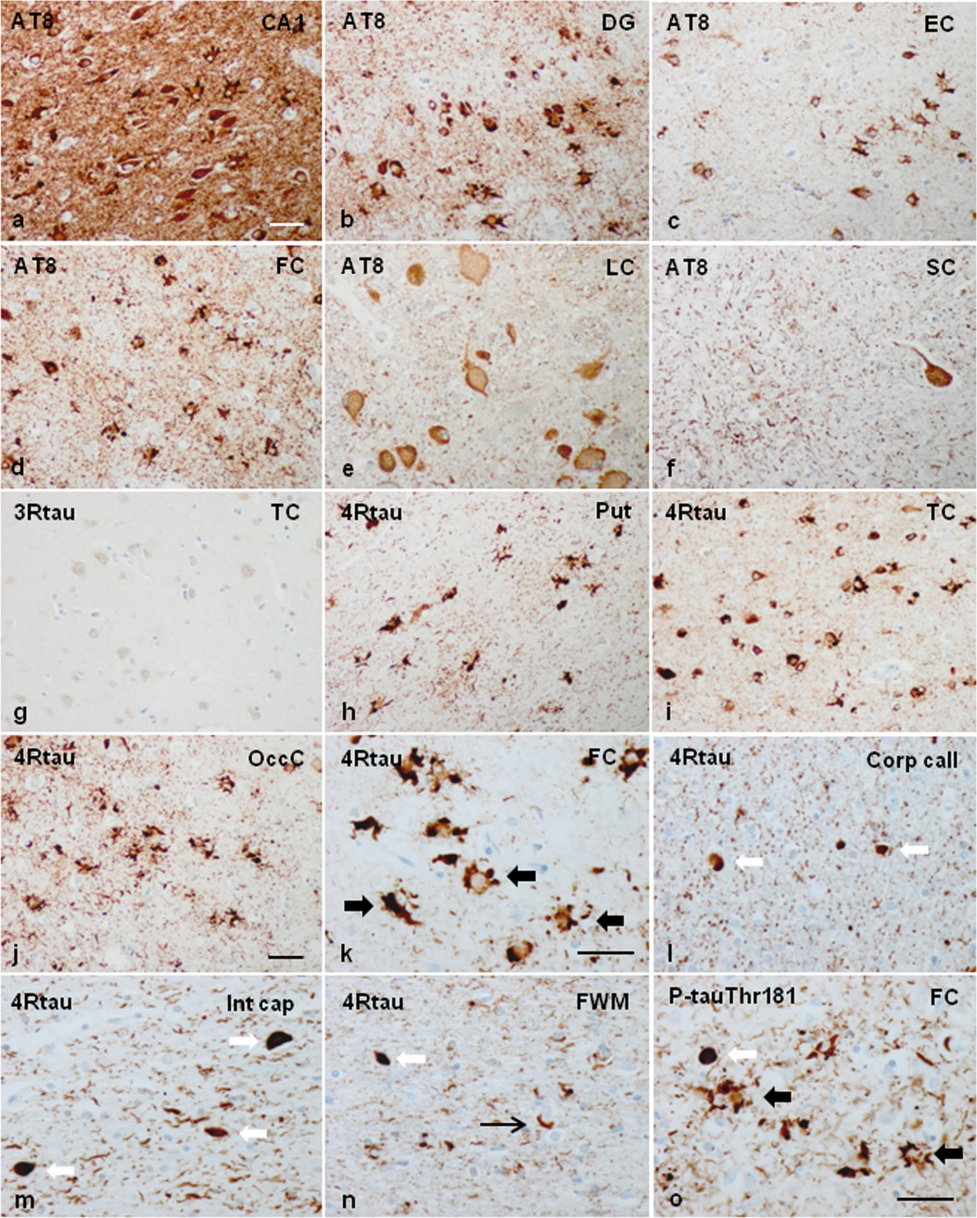

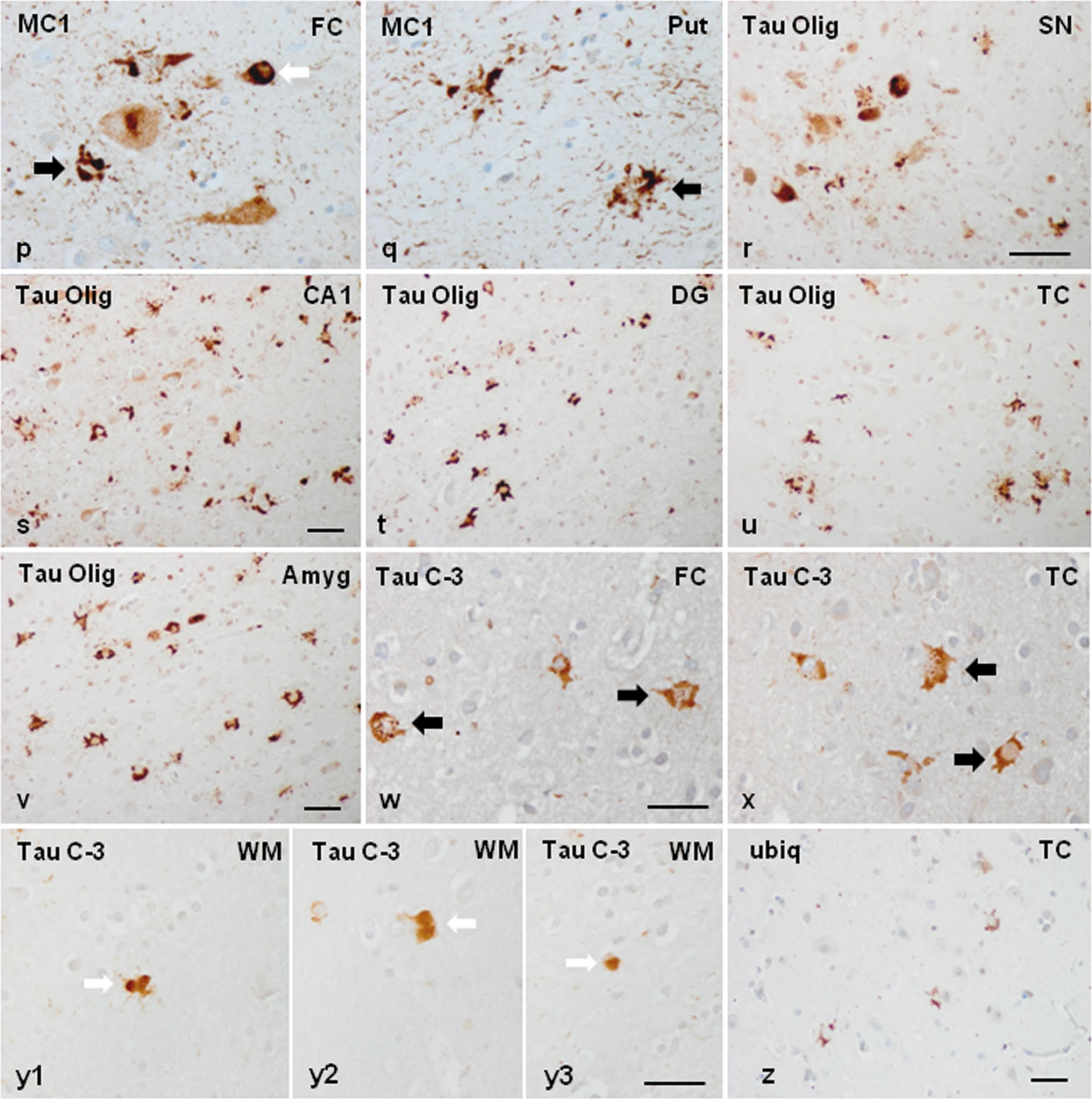
Representative neuropathological alterations in GGT linked to *MAPT* P301T mutation, case 1. Phospho-tau deposition identified with the antibody AT8 is seen in neurons and glial cells in the CA1 region of the hippocampus (CA1), dentate gyrus (DG), entorhinal cortex (EC), frontal cortex (FC), locus ceruleus (LC), and anterior horn of the spinal cord (SC) (**a**–**f**), among other regions. Tau deposits are not stained with anti-3Rtau antibodies (temporal cortex: TC) (**g**), but they are strongly immunoreactive with anti-4Rtau antibodies in all the assessed regions (here represented by the putamen: Put, temporal cortex: TC, and occipital cortex: OccC, frontal cortex, corpus callosum, internal capsule, and white matter of the frontal lobe) (**h**–**n**). At higher magnification, globular astrocytic inclusions (GAIs) are characterized by several oval-shaped or round peripheral phospho-tau-immunoreactive deposits in the proximal region of the astrocyte branches (**k**). Oligodendrocytes in the corpus callosum (Corp call), internal capsule (Int cap), and frontal subcortical white matter (FWM) have globular inclusions (GOIs), but some oligodendroglial inclusions are identical to coiled bodies (**i**–**n**). Phospho-tau immunoreactivity is also abundant in the neuropil of the grey matter and white matter. GAIs and GOIs are also stained with anti-PtauThr181 antibodies (**o**), and with MC1 antibodies which recognize abnormal tau conformation (as seen in the FC and Put) (**p**, **q**). Tau deposits in neurons and glial cells, including those in the hippocampus (CA1 region and dentate gyrus (DG), temporal cortex (TC), amygdala (Amyg), and nuclei of the brain stem such as the substantia nigra (SN) contain tau oligomers as revealed with the antibody tau-22 (**r**–**v**). Tau deposits, particularly in GAIs and GOIs, are also stained with Tau-C3, which recognizes tau truncated at Asp421, in most regions including the cerebral cortex (FC and TC) and white matter (WM) (**x, y1, y2, y3**). GAIs are labelled with thick black arrows, GOIs with thick white arrows and coiled bodies with thin arrows. Some neurons and glial cells are stained with anti-ubiquitin antibodies (**z**). Paraffin sections processed for immunohistochemistry slightly counterstained with haematoxylin; **a**–**j**, **p**–**e**, **h**–**j**, **s**–**v**, **z**, bar = 45 μm; **k**–**o**, **p**–**r**, **w**–**y3**, bar = 50 μm

Neurons, glial inclusions, and threads were stained with antibodies AT8, anti-P-tauThr181, anti-P-tauSer422, 4Rtau, and MC-1. Inclusions were Tau-C3 positive in a subpopulation of glial cells and neurons in two cases (case 1 and case 2) but rarely positive in the other two (cases 3 and 4). Inclusions in case 1 were particularly positive with the antibody tau-22 which recognizes oligomeric tau. Inclusions were negative with anti-3Rtau antibodies. Many oligodendroglial inclusions were positive with anti-ubiquitin and anti-p62 antibodies. The images in Fig. 1 illustrate neuropathological characteristics in case 1. Neuropathological findings in case 3 are shown in Supplementary Fig. 1.

**Fig. 2:**
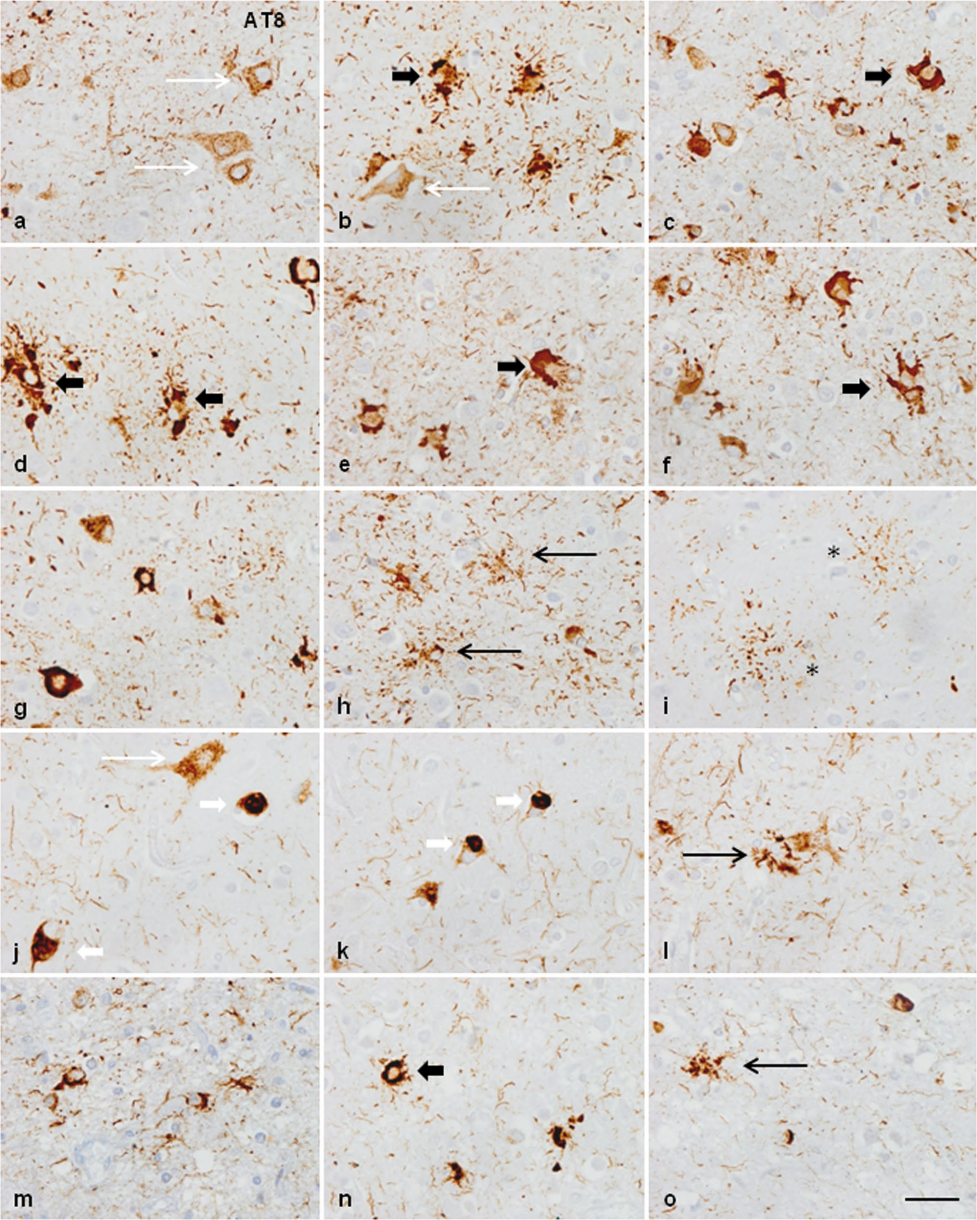
Characteristics of neuronal and astroglial phospho-tau deposits in the frontal cortex in GGT linked to *MAPT* P301T mutation; **a**–**i**: case 1; **j**–**o**: cases 3 and 4. In case 1, phospho-tau deposits, as revealed with the AT8 antibody, are finely granular in the cytoplasm, or form perinuclear halos, or globular tangles (long white arrow) (**a**, **b**, **d**, **g**). In cases 3 and 4, phospho-tau deposits are granular (long white arrow), globular, or round Pick-like bodies (short white arrow) (**j**, **k**). Astrocytic deposits in case 1 are typical GAIs with perikaryal globular structures or forming dense perinuclear inclusions of variable size consistent with immature stages (short black arrow) (**d**–**f**); together with astrocytes with longer cell and fine processes (long black arrow) (**h**), and astrocytic plaque-like structures (asterisk) (**i**). Astrocytic deposits in cases 3 and 4 show a predominance in astrocytes with longer cell processes (white arrows) (**l**, **m**, **o**) in addition to typical GAIs (short black arrows). Paraffin sections processed for AT8 immunohistochemistry and slightly counterstained with haematoxylin; bar = 25 μm

In addition to the individual variations in the distribution and magnitude of lesions, differential morphological details were also noted among cases. Neuronal phospho-tau deposits in case 1 were granular, perinuclear halos, dense deposits occupying all the cytoplasm, and tangles. Neuronal deposits in cases 2, 3 and 4 were granular, globular tangles, and round inclusions mimicking Pick bodies. Neuronal skein-like, and globular and dense inclusions in motor nuclei of the brain stem and spinal cord were observed mainly in case 1, but seldom encountered in case 3. Regarding astroglial deposits, GAIs in case 1 were perikaryal globular structures and coarse tufted-like deposits, sometimes forming rough dense perinuclear horse-shaped structures. In addition, astrocytes with longer radiating processes and structures similar to astrocytic plaques were also observed in case 1. GAIs with long processes rather than forming perikaryal structures were found in cases 2, 3, and 4 (Fig. 2). Many GAIs did not show mature morphology but variable numbers of perinuclear phospho-tau-positive deposits and short radiating fusiform or globular processes which were interpreted as immature forms [116].

Double-labelling immunofluorescence and confocal microscopy of the frontal cortex and white matter in case 1 using antibodies tau-C3, which recognizes tau truncated at aspartic acid 421, and anti-phospho-specific tau Thr181, showed that about 70% of phosphorylated tau-immunoreactive inclusions in astrocytes corresponding to GAIs, and oligodendrocytes corresponding to GOIs and coiled bodies, were immunostained with anti-Tau-C3 antibodies (data not shown). These numbers were similar to those already reported in sporadic GGT [40]. Similarly, about 70% of phospho-tau-immunoreactive inclusions in neurons, astrocytes, and oligodendrocytes, as revealed with AT8, were stained with tau-22 antibody.

Gallyas staining showed variable positivity in neurons, varying from tangle-like inclusions, dense diffuse staining, fine granular staining, and faint diffuse staining to more common Gallyas-negative tau-positive neurons (as revealed in consecutive sections immunostained with AT8 antibodies). GAIs were negative, although faint granular Gallyas-positive deposits were very rarely seen in the distal region of astrocytic processes or in the cytoplasm of a few astrocytes. In contrast, coiled bodies and GOIs were regularly positive in the four cases (Supplementary Fig. 5).

β-Amyloid and α-synuclein deposits were absent. TDP-43 immunohistochemistry did not disclose abnormal localization and distribution of this protein. Small blood vessel disease accompanied by mild status cribosus in basal ganglia was the only additional age-related lesion.

### mRNA expression of selected genes in frontal cortex and adjacent subcortical white matter in GGT linked to *MAPT* P301T mutation

Selected genes expressed in astrocytes and oligodendrocytes were assessed with RT-qPCR. In frontal cortex, *GFAP*, *ALDH1L1*, *YKL40,* and *GJA1* were significantly increased in GGT linked to *MAPT* P301T mutation when compared with controls (*p* = 0.0013, *p* = 0.000, *p* = 0.004, and *p* = 0.000, respectively). *MPC1* was significantly decreased (*p* = 0.006). *AQP4*, *SLC1A2*, *UCP4,* and *UCP5* mRNA expression did not differ in GGT when compared with controls. In contrast to astrocytes, oligodendrocyte- and myelin-related genes were not significantly altered in frontal cortex in GGT cases when compared with controls, with the exception of *NG2,* which was significantly increased in GGT (*p* = 0.02). However, a trend to reduced expression was noted after the examination of dot graphs (Supplementary Fig. 2).

In contrast to frontal cortex, the expression of astrocyte-related genes was preserved in the subcortical white matter in GGT cases when compared with controls, with the exception of a significant decrease in the expression of *MPC1* (*p* = 0.006). However, the expression of *OLIG1* and *OLIG2* was significantly reduced in the white matter in GGT (*p* = 0.004 and 0.02, respectively). In line with these findings, the mRNA expression of myelin-related genes *MBP*, *PLP1*, *CNP*, *MAG*, *MAL*, *MOG,* and *MOBP* was significantly decreased in GGT when compared with controls (*p* = 0.003, *p* = 0.001, *p* = 0.001, *p* = 0.008, *p* = 0.000, *p* = 0.004, and *p* = 0.009, respectively). Finally, mRNA expression of *SLC2A1* and *MCT1* was significantly reduced in GGT when compared with control cases (*p* = 0.04 and *p* = 0.015, respectively) (see Supplementary Fig. 3 for dot graphics).

### Expression of proteins related to astrocytes and oligodendrocytes in GGT linked to *MAPT* P301T mutation

**Fig. 3:**
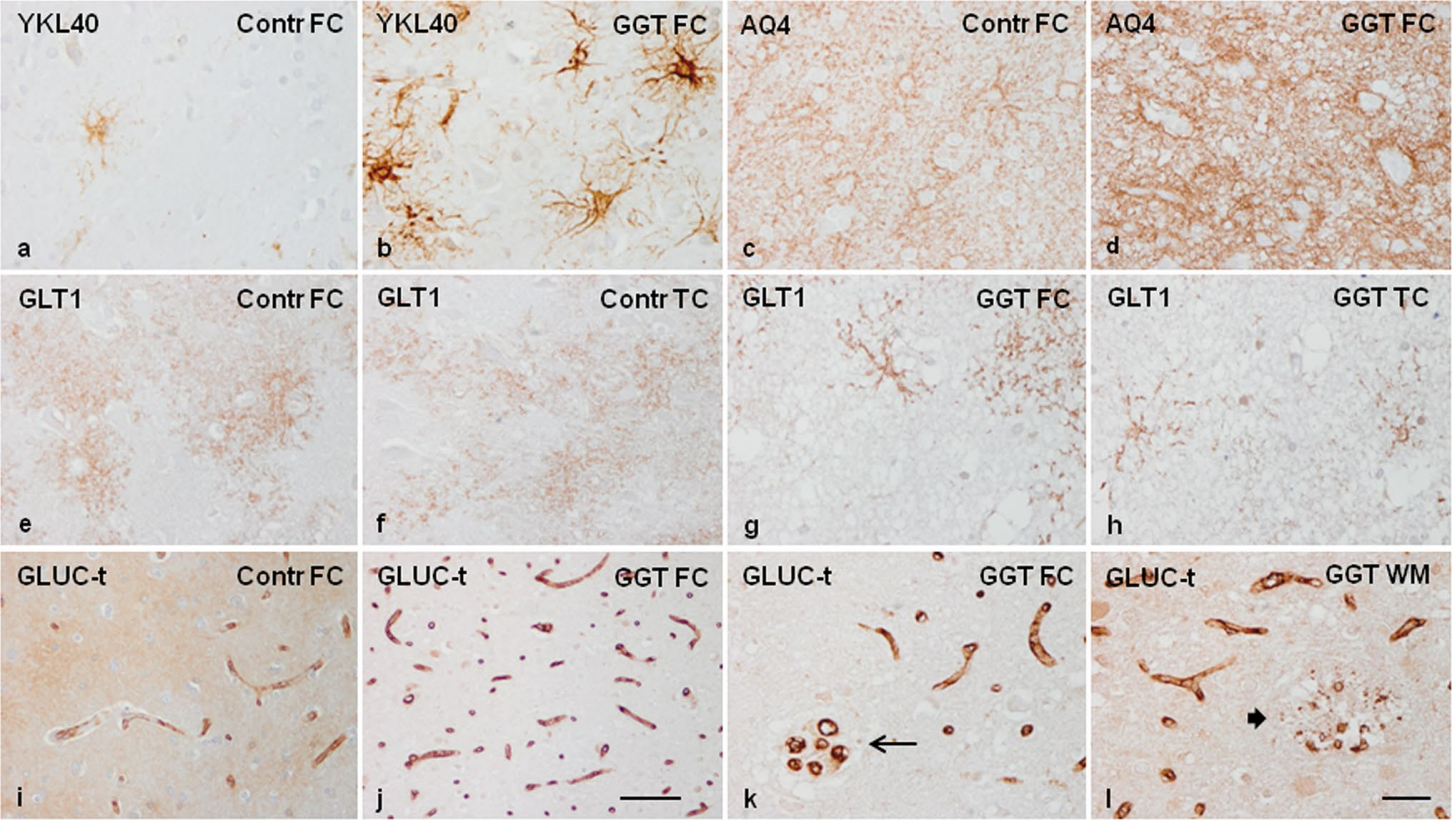
YKL40 is lightly expressed in very small subpopulations of astrocytes in the cerebral cortex in controls (Contr), but the number of YKL40-positive astrocytes, and the intensity of the immunoreactions per cell, are largely increased in cortical astrocytes in GGT linked to *MAPT* P301T mutation (**a**, **b**). AQ4 is also markedly increased in the cerebral cortex in GGT compared with controls (**c**, **d**). In contrast, the expression of the glutamate transporter GLT1 is reduced in the different regions of the cerebral cortex in GGT when compared with controls (**e**–**h**). The glucose transporter CLUC-t is expressed in the vessel wall of capillaries, and diffusely so in the neuropil in the cerebral cortex in control brains. The number of GLUC-t-immunoreactive capillaries is largely increased in the cerebral cortex in GGT linked to *MAPT* P301T mutation. This is accompanied by focal sprouting of capillaries in the cerebral cortex (thin arrow) and focal disruption of capillaries (thick arrow) in the frontal cortex and white matter in GGT (**i**–**l**). *FC* frontal cortex, *TC* temporal cortex, *WM* white matter. Case 1; paraffin sections processed for immunohistochemistry slightly counterstained with haematoxylin; **a**–**h**, **k**–**l**, bar = 45 μm; **i**, **j** bar = 100 μm

Astrocytic gliosis was accompanied by increased expression of YKL40 not only in the number of positive cells but also in the density of the reaction in a subpopulation of astrocytes in GGT (Fig. 3a, b). Aquaporin AQ4, encoded by AQP4, was expressed in astrocyte processes including podocytes around blood vessels in normal conditions; however, AQ4 immunoreactivity was much denser, giving rise to a jammed meshwork in GGT (Fig. 3c, d). Glutamate transporter GLT1 (excitatory amino acid transporter 2: EAAT2), encoded by *SLC1A2*, was also localized in the branches of astrocytes, forming a delicate net in grey matter of control brains; this pattern was markedly altered in affected areas of the cerebral cortex in GGT in which only scattered astrocytes presented GLT1 immunoreactivity (Fig. 3e–h). Solute carrier family 2 member 1: glucose transporter GLUC-t (here named in this way and not as GLT1 to avoid confusion with the glutamate transporter) encoded by SLC2A1, was mainly expressed in the vessel wall of capillaries but also as a diffuse component of the neuropil in control brains. GLUC-t expression was abnormal in the cerebral cortex and white matter in GGT cases (particularly in case 1) due to the increased number of capillaries and reduced immunoreactivity in the neuropil (Fig. 3i, j). Quantification of capillaries in the frontal cortex revealed a significant increase when compared with controls (control: 10.92 ± 0.28 vs. 47.7 ± 1.44; *p* = 0.000). Increased number of capillaries can be interpreted as the result of cortical atrophy, but the presence of focal capillary sprouting and foci of disrupted capillaries in grey matter and white matter points to a possible primary disorder of capillaries in GGT (Fig. 3k, l).

**Fig. 4:**
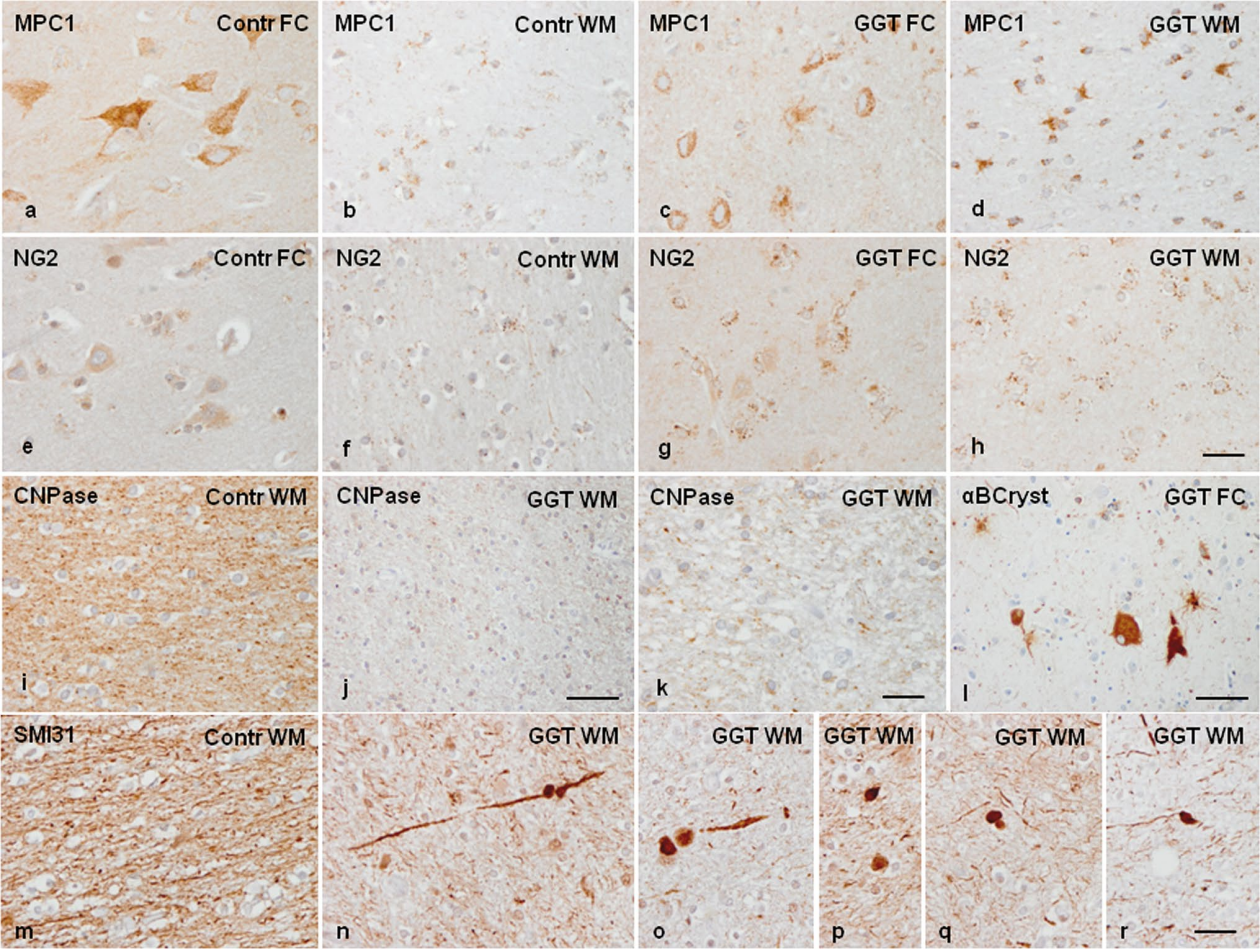
GGT linked to *MAPT* P301T mutation. Mitochondrial pyruvate carrier 1 (MPC1) is expressed in neurons in the cerebral cortex and in a subpopulation of glial cells in the frontal cortex (FC) and white matter (WM) in control (Contr) and GGT cases. MPC1 immunoreactivity is markedly reduced in neurons (decreased numbers of intracytoplasmic granules), but dramatically augmented in glial cells resembling astrocytes in the white matter in GGT when compared with controls (**a**–**d**). NG2 immunoreactivity is found in a subset of glial cells, mainly satellite cells, in the cerebral cortex, and in a number of glial cells in the white matter. The number of glial cells with NG2-immunoreactive granules is higher in the cerebral cortex and white matter in GGT cases when compared with controls (**e**–**h**). CNPase, used as a marker of myelin, is markedly decreased in the white matter in GGT when compared with controls, with disruption of remaining fibres in the white matter seen in GGT cases (**i**–**k**). αB-crystallin immunoreactivity decorates ballooned neurons and a subpopulation of astrocytes and oligodendrocytes in GGT (**l**). SMI31 immunohistochemistry reveals reduced numbers of nerve fibres, varicosities, and axonal ballooning, and SMI31-immunoreactive inclusions in the white matter in GGT cases (**n**–**r**) when compared with the white matter in controls (**m**). Case 1; paraffin sections processed for immunohistochemistry slightly counterstained with haematoxylin; **a**–**h**, **k**, bar = 45 μm; **i**, **j**–**l**, **m**–**r**, bar = 100 μm

Mitochondrial pyruvate carrier 1, encoded by *MPC1*, was expressed mainly in neurons in the cerebral cortex and in a subpopulation of glial cells in the grey and, particularly, the white matter, where it was identified as small confluent cytoplasmic granules. MPC1 immunoreactivity was markedly reduced in individual neurons in the cerebral cortex in GGT when compared with controls, but MPC1 immunoreactivity was markedly increased in glial cells resembling astrocytes because of the distribution of their branches in the white matter of the same cases (Fig. 4a–d).

NG2 immunoreactivity was recognized as small granules in the cytoplasm of a subpopulation of glial cells, mainly satellite cells, in the cerebral cortex, and in a number of glial cells in the white matter in control brains. The number of glial cells positive with the NG2 antibody was greater in the cerebral cortex and white matter in GGT cases when compared with controls (Fig. 4e–h). CNPase, used as a marker of myelin, was expressed in the white matter and intra-cortical myelin fibres; the number of CNPase-immunoreactive fibres was markedly reduced in the white matter in GGT cases, and this loss was accompanied by disruption of the remaining fibres (Fig. 4i–k). Similar results were obtained with anti-PLP1 and anti-MBP antibodies (data not shown). Finally, in order to reveal axonal damage, SMI31 immunohistochemistry revealed dramatic axonal disruption, axonal varicosities, and axonal spheroids along nerve fibres in the white matter (Fig. 4m–r).

To analyse whether altered protein expression of different proteins was linked to phospho-tau deposition within the same cells, selected proteins were assessed with double-labelling immunofluorescence and confocal microscopy. Ballooned neurons, many reactive astrocytes, and scattered oligodendrocytes contained αB-crystallin (Fig. 4l), but double-labelling immunofluorescence disclosed no co-localization of phospho-tau (antibody P-tauThr181) or αB-crystallin in the vast majority of neurons and astrocytes containing one of these proteins (data not shown), in agreement with previous data detailed elsewhere [85]. A similar situation occured in relation to YKL40 since it is mainly expressed in reactive astrocytes independently of the presence of protein aggregates (data not shown), as observed in many other neurodegenerative diseases [33]. In contrast, phospho-tau deposition in astrocytes dramatically modified the distribution of the cytoskeletal protein GFAP in GGT as revealed by double-labelling immunohistochemistry and confocal microscopy (data not shown) in agreement with previous observations in sporadic GGT and other tauopathies [33, 34].

**Fig. 5:**
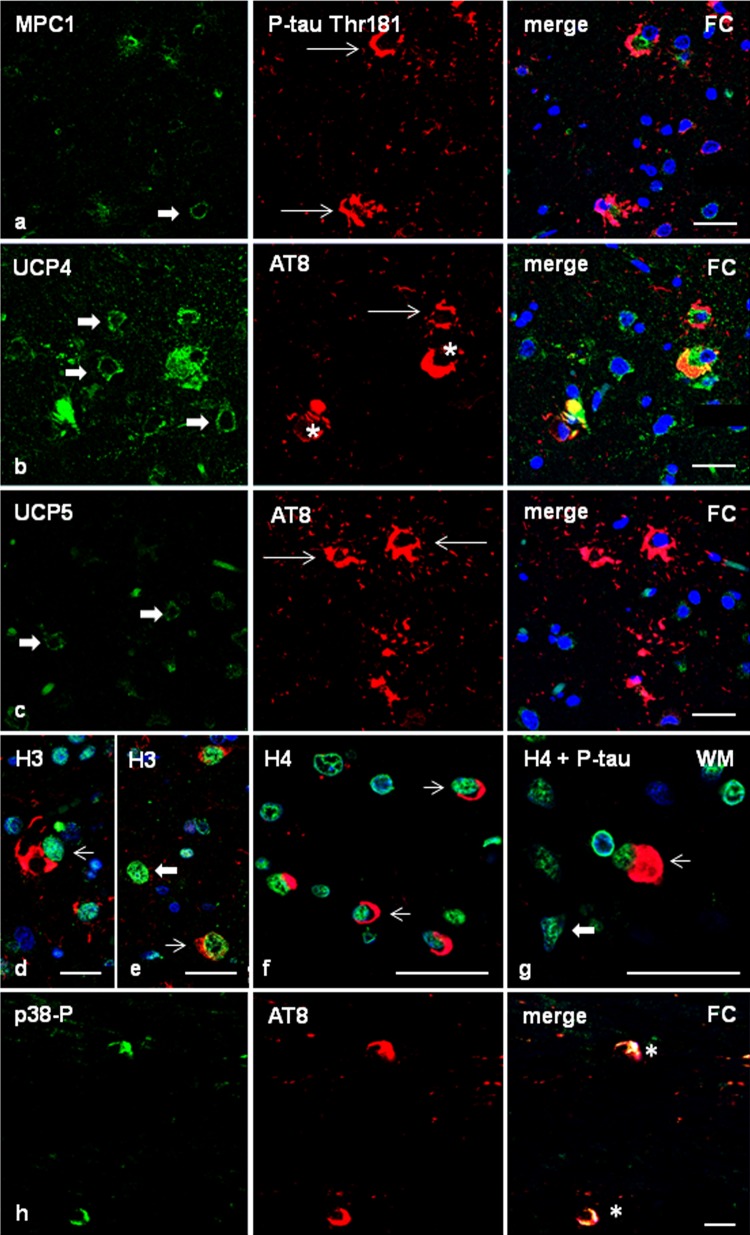
Frontal cortex in GGT linked to *MAPT* P301T mutation (case 1). Double-labelling immunofluorescence and confocal microcopy of the frontal cortex (FC) in case 1 using antibodies anti-MPC1 (**a**), UCP4 (**b**), or UCP5 (**c**) (green), and anti-P-tau Thr181 or AT8 (red). MPC1 is expressed equally in astrocytes with tau deposits (thin arrows) and without tau deposits (thick arrow) (**a**). Similarly, UCP4 is found in astrocytes bearing phosphorylated tau (thin arrows) and in cells without tau deposits (thick arrows) (**b**). In contrast, UCP5 is absent in GAIs deposits (thin arrows) when compared with cells without tau deposits (thick arrows) (**c**). Nuclei are counterstained with DRAQ5™ (blue). Paraffin sections, **a**–**c**, bar = 30 μm. **d**–**g** Expression of histones in frontal cortex and white matter in GGT (case 1). H3K9me2 immunoreactivity is expressed equally in cells with (thin arrows) and without (thick arrows) phosphorylated tau deposits (P-tauThr181 antibody) (**d**, **e**). Similarly, H4K12ac immunoreactivity is expressed equally in oligodendrocytes with coiled bodies (**f**) and globular inclusions (**g**) (AT8 antibody) (thin arrows), and in oligodendroglia cells without tau inclusions (thick arrows). Nuclei are counterstained with DRAQ5™ (blue). Paraffin sections, **d** bar = 20 μm; **e**–**g** bar = 30 μm. **h** phosphorylated p38 Thr180-Tyr182 (p38P) co-localizes with AT8-immunoreactive deposits (asterisk) in frontal cortex in GGT. Paraffin section without nuclear counterstaining, bar = 10 μm

In order to learn about the impact of phospho-tau deposition in astrocytes containing hyper-phosphorylated tau deposits, double-labelling immunofluorescence to mitochondrial pyruvate carrier 1 (MPC1) and confocal microscopy disclosed similar MPC1 immunoreactivity in phospho-tau-containing glial cells and in glial cells without phospho-tau deposits in the frontal cortex of GGT cases (Fig. 5a). Similarly, the expression of mitochondrial uncoupling protein 4 (UCP4) was similar in glial cells with and without phospho-tau deposits. Robust UCP4 immunoreactivity was also observed in neurons containing phospho-tau deposits (Fig. 5b). In contrast, mitochondrial uncoupling protein 5 (UCP5) immunoreactivity was almost absent in GAIs when compared with neighbouring glial cells without phospho-tau deposits (Fig. 5c).

Double-labelling immunofluorescence and confocal microscopy to H3K9me2 and H4K12ac, and phospho-tau antibodies (P-tauThr181 and AT8, respectively), showed similar histone H4K12ac and H3K12ac immunoreactivity in the nuclei of cells with and without phospho-tau depositions (Fig. 5d–g).

Finally, double-labelling immunofluorescence and confocal microscopy to phosphorylated kinase p38 (p38-P Thr180-Tyr182) and AT8 disclosed p38-P immunoreactivity restricted to neurons and glial cells containing phospho-tau deposits in about 70% of the population bearing tau deposits (Fig. 5h).

### Neuroanatomical proteostatic imbalance in GGT linked to *MAPT* P301T mutation

**Fig. 6:**
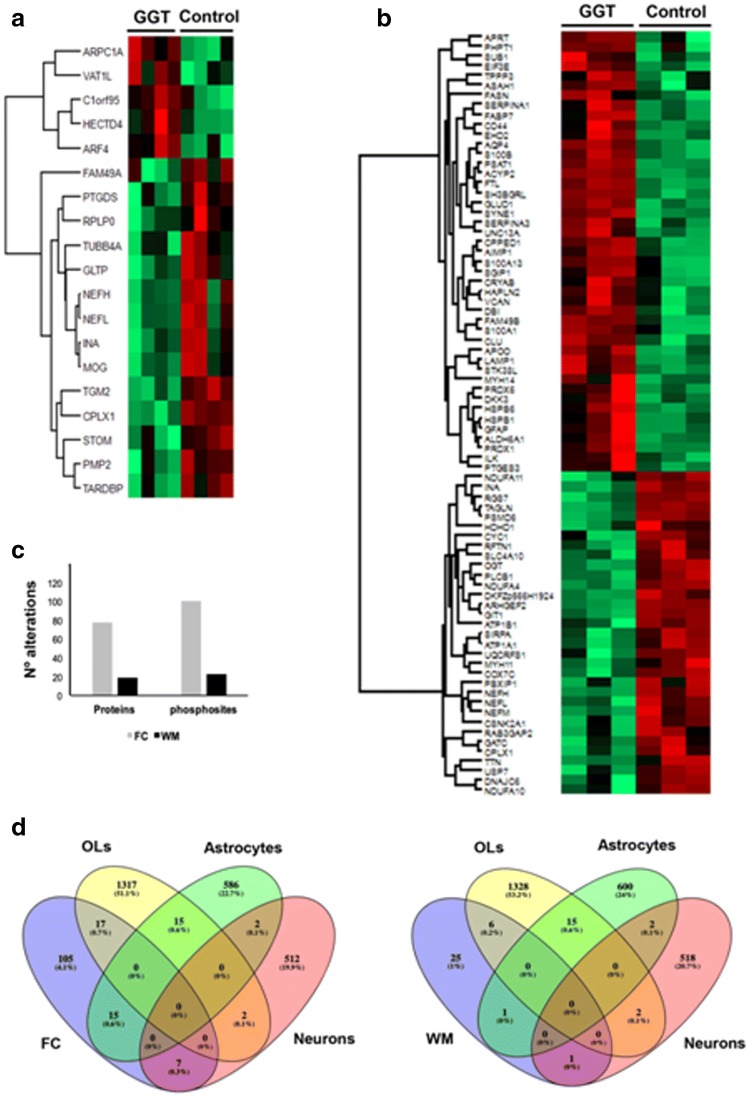
Frontal cortex (FC) and white matter (WM) proteomic disturbances in GGT linked to *MAPT* P301T mutations compared with controls. Heat map represents the differentially expressed proteins between GGT cases and controls in WM (**a**) and FC (**b**); red and green, up-regulated and down-regulated proteins, respectively. **c** Number of proteome and phosphoproteome alterations in both areas. **d** Specific-cell-type enrichment of proteostatic alterations detected in FC and subcortical WM

**Fig. 7:**
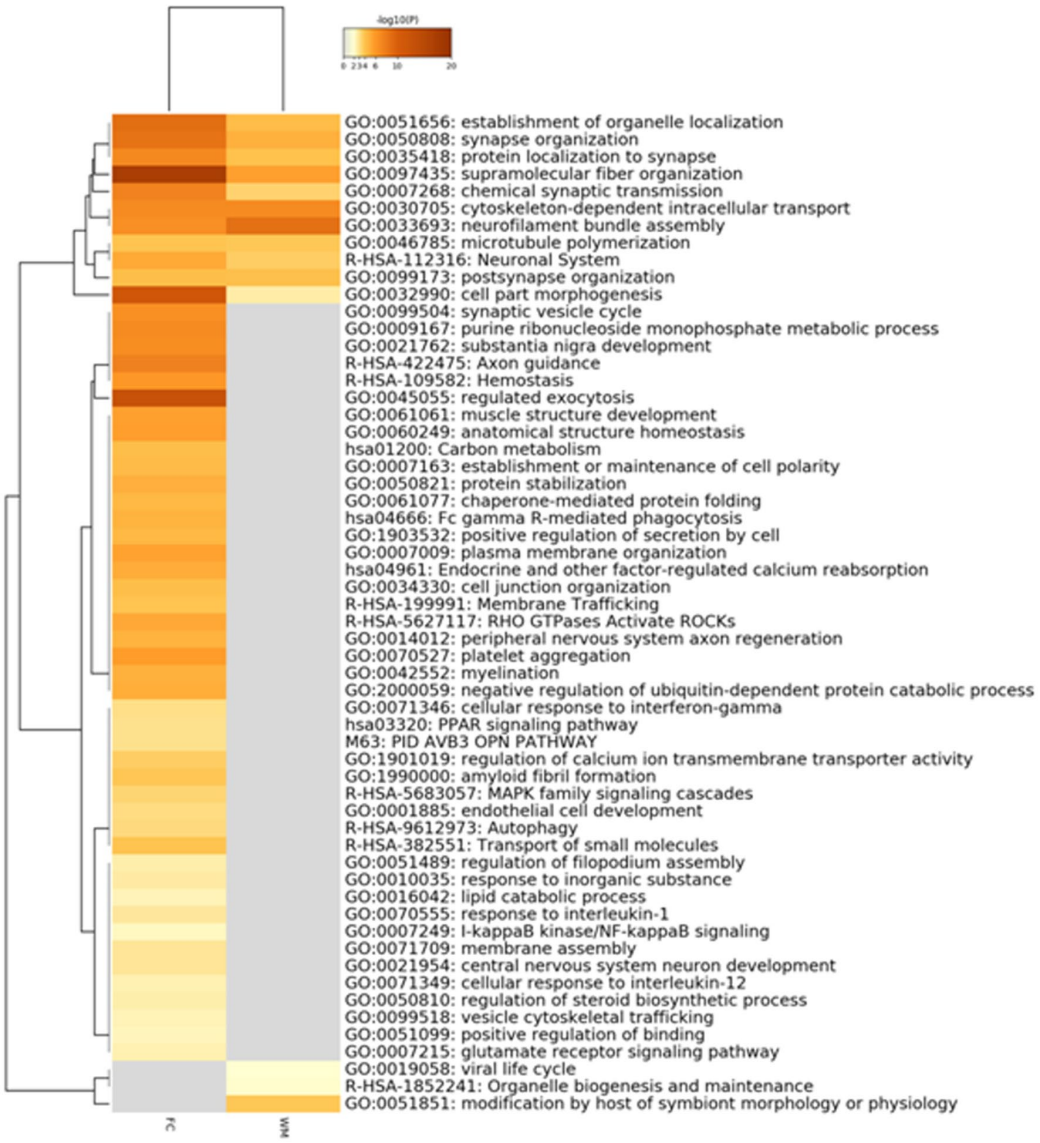
Enriched ontology clusters across frontal cortex (FC) and white matter (WM) differential (phospho)proteomes in GGT linked to *MAPT* P301T mutation. This analysis was made using Metascape. After the identification of all statistically enriched terms (GO/KEGG terms, canonical pathways, hall mark gene sets), cumulative hypergeometric *p* values and enrichment factors were calculated and used for filtering. Remaining significant terms were then hierarchically clustered into a tree based on Kappa-statistical similarities among their gene memberships. Then, 0.3 kappa score was applied as the threshold to cast the tree into term clusters. The term with the best *p* value within each cluster was selected as its representative term and displayed in a dendrogram. The heat map cells are coloured by their *p* values; white cells indicate the lack of enrichment for that term in the corresponding gene list

With the aim of gaining insight into the proteostatic dearrangement present in GGT, mass-spectrometry-based quantitative proteomics was applied at the level of frontal cortex and subcortical white matter. Proteome monitoring revealed 78 and 19 differentially expressed proteins in frontal cortex and white matter, respectively, in GGT cases with respect to controls (Supplementary Tables 1 and 2 and Fig. 6a, b), uncovering 4 common differentially underexpressed proteins in both areas (neurofilament light, neurofilament heavy, internexin neuronal intermediate filament protein α, and complexin 1: NEFL, NEFH, INA and CPLX1, respectively). Moreover, phosphoproteome profiling revealed 101 and 23 differential phosphopeptides (mapping to 74 and 15 proteins) corresponding to frontal cortex and white matter, respectively. Changes in the phosphorylation state observed in 8 cortical proteins (aquaporin 4, glial fibrillary acidic protein, heat shock protein family B (small) member 1, heat shock protein family B (small) member 6, neurofilament light, neurofilament heavy, neurofilament medium, and solute carrier family 4 member 10: AQ4, GFAP, HSPB1, HSPB6, NEFH, NEFL, NEFM, SLC4A10***,*** respectively), and in one white matter protein (NEFH), may be associated with total protein variations. Three cytoskeletal-associated phosphoproteins were commonly deregulated in both structures: microtubule-associated protein tau, microtubule-associated protein A, and microtubule-associated protein B (MAPT, MAP1A, and MAP1B, respectively) (Supplementary Tables 1 and 2). To characterize the metabolic modulation in frontal and subcortical white matter, differential proteomic and phosphoproteomic maps (Fig. 6c) were merged and functionally analysed. A cell-type enrichment analysis across frontal cortex and white matter differential datasets was performed using cell-type protein marker lists derived from four purified brain cell types: neuron, astrocyte, microglia, and oligodendrocyte [105, 137]. As shown in Fig. 6d, part of the frontal cortex alterations corresponded to oligodendrocytes (12%), astrocytes (10.5%), and neurons (5%) (Supplementary Table 3). In the case of white matter, 18% of proteostatic alterations were specific to oligodendrocytes, and 3% of astrocytic and neuronal nature (Fig. 6d, Supplementary Table 3). All these data shed new light on the molecular disturbances that accompany tau deposition across each cellular homeostasis in GGT. To amass a more detailed description of the molecular mechanisms involved in frontal cortex and white matter in GGT, subsequent analyses were performed to explore the differential (phospho)proteome distributions across specific pathways/biofunctions. Axon guidance, exocytosis, chaperone-mediated protein folding, and myelination were part of the significantly over-represented dysregulated processes in the frontal cortex (Fig. 6, Supplementary Table 4). Interestingly, our data point up a common deregulation of specific protein clusters related to microtubule polymerization and synaptic transmission (Fig. 7, Supplementary Table 4). A deep synaptic ontology analysis (Table 3, Supplementary Table 5) revealed that protein derangements occur at the presynaptic and postsynaptic levels, being partially involved in the structural integrity of the synapse in GGT cases linked to *MAPT* P301T mutation.

**Table 3:** Synaptic ontologies across frontal cortex and white matter differential (phospho)proteomes

	Proteins	*p* value	*q* value
Subcortical white matter: synaptic ontologies			
Postsynaptic intermediate filament cytoskeleton	4	3.64e−10	1.09e−9
Postsynapse	7	1.02e−4	1.52e−4
Presynapse	4	0.0122	0.0122
Structural constituent of postsynaptic intermediate filament cytoskeleton	3	6.07e−8	2.43e−7
Postsynaptic modulation of chemical synaptic transmission	4	1.52e−7	3.03e−7
Process in the synapse	7	5.95e−4	7.93e−4
Synapse organization	4	1.96e−3	1.96e−3
Frontal cortex: synaptic ontologies			
Postsynapse	23	5.18e−10	3.63e−9
Postsynaptic cytoskeleton	7	1.74e−9	8.12e−9
Presynapse	20	2.45e−9	8.57e−9
Postsynaptic intermediate filament cytoskeleton	4	1.30e−7	3.63e−7
Postsynaptic density, intracellular component	4	3.35e−4	7.81e−4
Presynaptic cytosol	3	1.69e−3	3.38e−3
Presynaptic active zone	4	5.93e−3	0.0104
Synaptic vesicle	4	0.0125	0.0159
Postsynaptic specialization	7	0.0113	0.0159
Postsynaptic membrane	4	0.0121	0.0159
Postsynaptic density	6	0.0145	0.0169
Synaptic vesicle membrane	3	0.0300	0.0323
Structural constituent of synapse	8	9.76e−10	7.32e−9
Structural constituent of postsynapse	7	2.95e−9	1.48e−8
Chemical synaptic transmission	11	3.27e−8	1.23e−7
Synapse organization	14	1.00e−7	3.01e−7
Synaptic vesicle cycle	10	1.81e−6	4.37e−6
Postsynaptic modulation of chemical synaptic transmission	5	2.04e−6	4.37e−6
Process in the presynapse	11	4.99e−6	8.32e−6
Structural constituent of postsynaptic intermediate filament cytoskeleton	3	4.81e−6	8.32e−6
Modulation of chemical synaptic transmission	6	6.63e−5	9.95e−5
Process in the postsynapse	8	1.23e−4	1.68e−4
Synaptic vesicle exocytosis	5	3.02e−4	3.78e−4
Regulation of postsynaptic membrane neurotransmitter receptor levels	5	1.55e−3	1.79e−3
Regulation of postsynaptic neurotransmitter receptor endocytosis	3	1.69e−3	1.81e−3
Synaptic vesicle endocytosis	3	7.68e−3	7.68e−3

The protein count column shows the number of proteins that are annotated in SynGO [65] against each term. The “brain expressed” background set was selected, containing 18,035 unique genes in total of which 1104 overlap with SynGO annotated genes. For each ontology term, a one-sided Fisher exact test was performed to compare differential datasets and the “brain expressed” background set. The result is shown in the “*p* value” column. To find enriched terms within the entire SynGO ontology, a multiple testing correction using false discovery rate (FDR) was applied (*q* value column)

### Other GGT cases

#### Sporadic GGT

The neuropathological study of the sGGT case revealed severe frontotemporal atrophy with marked enlargement of the lateral ventricles and atrophy of the perisylvian region, reduced white matter, marked atrophy of the corpus callosum, and moderate atrophy of the caudate and putamen, as well as the thalamus. The microscopical study revealed marked neuron loss and astrocytic gliosis in the cerebral cortex. Phospho-tau-immunoreactive neurons were seen in the cerebral cortex (particularly in the frontal cortex), hippocampus, diencephalon, substantia nigra, reticular formation, and motor nuclei of the brainstem. In addition to neurons, accumulation of phospho-tau was present in astrocytes forming GAIs (which were less abundant in the frontal cortex when compared with the other GGT cases assessed in this series), in oligodendrocytes forming GOIs (very abundant in the frontal white matter) and, less commonly, coiled bodies. Phospho-tau deposits in neurons had variegated morphologies including small granular deposits, round or oval-shaped cytoplasmic inclusions, coarse granular cytoplasmic deposits, dense inclusions occupying all the cytoplasm, and skein-like inclusions in motor nuclei of the brainstem; tangles were not observed. Similar skein-like deposits were found in the motor neurons of the spinal cord. Phospho-tau deposits in astrocytes were scarce and reminiscent of small GAIs. In contrast, GOIs were often large, globose, and often bilobulated, but coiled bodies were very rare in the white matter (see case 1, [38]). Phospho-tau inclusions were positive with antibodies against 4Rtau but negative with antibodies against 3Rtau excepting a minority of glial deposits. Tau deposits in neurons, astrocytes, and oligodendrocytes were stained with antibodies against P-tauThr181, P-tauSer422, AT8, and MC-1; a subpopulation of oligodendroglial inclusions was stained with anti-Tau-C3 and anti-ubiquitin antibodies, but neurons were only very rarely so, as detailed elsewhere [40]. GOIs were also stained with antibodies against oligomeric tau (data not shown). GAIs were Gallyas-negative and GOIs Gallyas-positive (Supplementary Fig. 5).

#### Familial GGT linked to *MAPT* K317M mutation

**Fig. 8:**
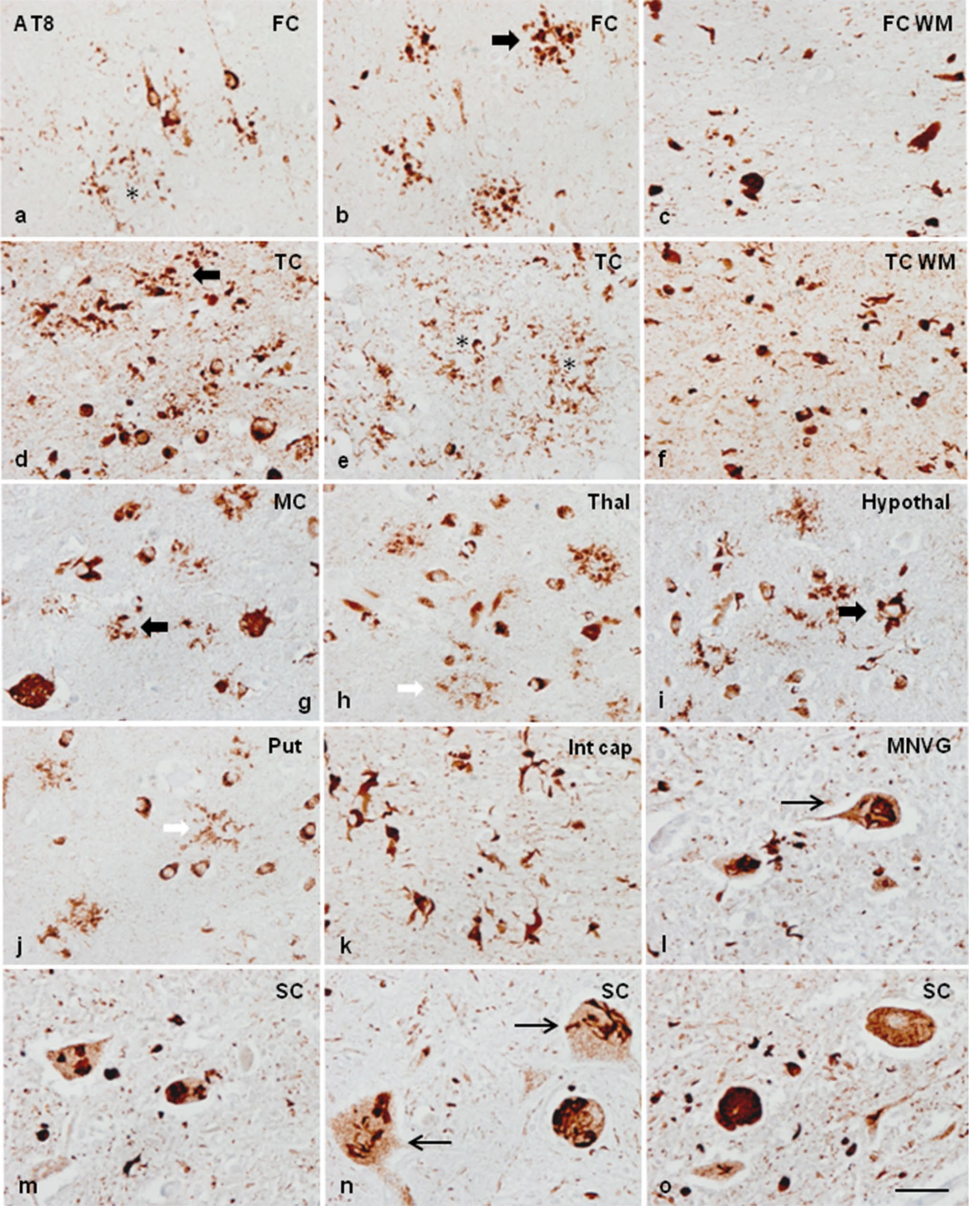
Representative neuropathological alterations in GGT linked to *MAPT* K317M mutation. Phosphorylated tau deposition identified with the antibody AT8 is found in different types of neurons and glial cells in various regions including frontal cortex (FC), and frontal cortex white matter (FWM), temporal cortex (TC) and white matter (TCWM), motor cortex (MC), thalamus (Thal), hypothalamus (Hypothal), putamen (Put), internal capsule (int cap), motor nucleus of the vagus nerve (MNVG), and anterior horn of the spinal cord (SC) of one affected brother. Neuronal deposits form small granules in the cytoplasm (**a**, **o**), dense inclusions (**d**, **m**), globose tangles (**g**, **o**), and skein-like inclusions in the motor nuclei of the brainstem (**l**) and motor neurons of the spinal cord (**m**–**o**) (black thin arrows). Astroglial inclusions are GAIs of variable morphology including astrocytes with perikaryal globular inclusions (black thick arrows) (**b**, **d**, **g**, **i**), astrocytes with longer processes (**h, j**) (white short arrows), and astrocytic plaque-like structures (asterisks) (**a**, **e**). Oligodendroglial inclusions are GOIs and coiled bodies (**c**, **f**, **k**). Paraffin sections processed for immunohistochemistry slightly counterstained with haematoxylin, bar = 40 μm

The neuropathological study of GGT linked to *MAPT* K317M mutation [40, 135] showed marked atrophy of the frontal and temporal lobes, and depigmentation of the substantia nigra. On microscopical examination, the present information complements and offers additional details to the original report [135]. Severe neuron loss and spongiosis was found in the frontal and temporal cortices, primary motor cortex, substantia nigra, and motor nuclei of the medulla oblongata. The corticospinal tracts showed severe demyelination at the level of the bulbar pyramidal tracts. The spinal cord was not available for study in this case, but neuropathological examination of the spinal cord of the subject’s affected brother showed severe demyelination and axonal loss in the pyramidal tracts and loss of motor neurons in the anterior horn of the spinal cord [135]. Astrocytic gliosis, as revealed with GFAP immunohistochemistry, was marked in all these regions. YKL40-immunoreactive astrocytes were numerous, and they were of huge size and with robust cellular processes in the cerebral cortex. Tau immunohistochemistry revealed massive deposition of phospho-tau in neurons, astrocytes, and oligodendrocytes in all the above-mentioned regions and in the hippocampus, entorhinal cortex, thalamus, striatum, subthalamus, hypothalamus, mammillary bodies, and nuclei of the brainstem. Phospho-tau deposits in neurons were variable in morphology and manifested as small diffuse granular cytoplasmic deposits, large aggregates, and globular tangles. Phospho-tau deposits in neurons of the motor nucleus of the vagus nerve showed skein-like and globular inclusions. Phospho-tau deposits in the spinal cord of the brother showed various types of neuronal deposits including granular deposits, filamentous and skein-like inclusions, globular bodies, and, rarely, globose tangles. Phospho-tau deposits in astrocytes had heterogenous morphology including abundant GAIs with perikaryal globular structures, rare tufted-like astrocytes, rare astrocytic plaques, astrocytes with radiating processes, and astrocytes with mixed morphologies. Phospho-tau deposits in oligodendrocytes were GOIs and coiled bodies. GOIs were abundant in the white matter and had marked polymorphism, often presenting coarse globular processes in addition to large, bizarre coiled bodies. All the inclusions were stained with 4Rtau antibodies but not with antibodies against 3Rtau, with the exception of a very few oligodendroglial inclusions (Fig. 8). Tau deposits in neurons and glial cells were stained with antibodies against P-tauThr181, P-tauSer422, AT8, and MC-1 and, less commonly, with Tau-C3, as detailed elsewhere [40], as well as with antibodies against oligomeric tau (data not shown); many tau inclusions were also ubiquitinated.

Gallyas staining in this case showed the same changes as those seen in GGT linked to *MAPT* P301T mutation. Particular features in this case were numerous Gallyas-positive threads in the cerebral cortex and bizarre globular inclusions in addition to GOIs. GAIs were Gallyas-negative, but astrocytes with perinuclear Gallyas-positive small granular inclusions were seldom observed (Supplementary Fig. 5).

### Expression of proteins related to astrocytes and oligodendrocytes in sGGT and GGT linked to *MAPT* K317M mutation

Only the expression of a few proteins was analysed in the frontal cortex in these cases due to the limited availability of samples.

In sGGT, AQ4 immunoreactivity was reduced when compared with controls, excepting AQ4 preservation around blood vessels. GLUC-t was decreased in the neuropil (Supplementary Fig. 6).

In GGT linked to *MAPT* K317M mutation, GLUC-t, GLT1, and AQ4 immunoreactivity was reduced in the neuropil when compared with controls (Supplementary Fig.  6).

GLUC-t immunoreactivity decorated the capillaries (Supplementary Fig. 6). The number of GLUC-t-immunoreactive capillaries per area was significantly increased in sGGT 36.6 ± 1.36) and GGT linked to *MAPT* K317M mutation (40.4 ± 3.50) when compared with controls (10.92 ± 0.28) (*p* = 0.000).

### Biochemical and morphological characteristic of sarkosyl-insoluble fractions of human brain homogenates in GGT cases linked to *MAPT* P301T mutation, sGGT, and GGT linked to *MAPT* K317M

**Fig. 9:**
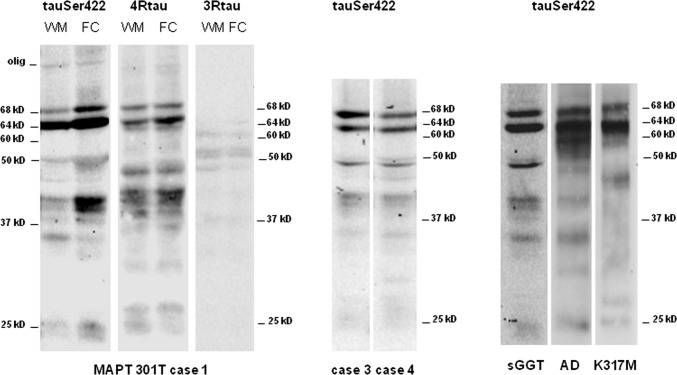
Gel electrophoresis and western blotting of sarkosyl-insoluble fractions from the frontal cortex (FC) and subcortical frontal white matter (WM) in GGT cases linked to *MAPT* P301T, FC in sGGT, and FC in GGT linked to *MAPT* K317M mutations, using P-tauSer422 (tauP422) antibody. Western blots of the WM and FC in GGT linked to *MAPT* P301T case 1 show two strong bands of 68 kDa and 64 kDa and weaker bands of 50 kDa and 40 kDa, in addition to two lower bands of about 25 kDa and 22 kDa. The intensity of a band of 35 kDa is higher in the WM when compared with the FC run in parallel. A similar band pattern is found with anti-4Rtau antibodies. In contrast, a very weak signal, if any, is obtained with the anti-3Rtau antibody. Western blots of sarkosyl-insoluble fractions of the frontal cortex from GGT linked to *MAPT* P301T mutation, cases 3 and 4 stained with anti-P-tauSer422 antibodies show two bands of 68 kDa and 64 kDa, a doublet of about 50 kDa, weak bands of about 40 kDa, one band of about 35 kDa, and a weak band of about 25 kDa. Sarkosyl-insoluble fractions of frontal cortex homogenates from sGGT case blotted with anti-P-tauSer422 antibodies show bands with a pattern similar to cases 3 and 4, but with more defined bands of about 45 kDa and 35 kDa, and a lower band of about 20 kDa. Western blots of sarkosyl-insoluble fractions of the frontal cortex from GGT linked to *MAPT* K317M mutation show two bands of 68 kDa and 64 kDa, a smear of about 60 kDa, smears of about 40 kDa and below, and a weak band of about 25 kDa. For comparative purposes, western blots of sarkosyl-insoluble fractions of the frontal cortex from one case of Alzheimer’s disease stage VI of Braak and Braak show three bands of 68 kDa, 64 kDa, and 60 kDa, lower bands of about 45 kDa and 35 kDa, and one lower band of about 25 kDa

Sarkosyl-insoluble fractions of frontal cortex and subcortical white matter homogenates from case 1, GGT linked to *MAPT* P301T mutation, when blotted with anti-P-tauSer422 showed similar band patterns in the frontal cortex and subcortical white matter processed in parallel. Bands of 68 kDa and 64 kDa were characteristic of 4Rtaupathy; in addition, lower bands of 50 kDa, several bands of about 40 kDa, a band of about 35 kDa, and two bands of 25 kDa and 22 kDa were seen in the white matter and frontal cortex. The intensity of the bands was higher in the grey matter than in the subcortical white matter except for the band of about 35 kDa, suggesting minor differences between cerebral cortex and underlying white matter regarding tau species. Western blots processed with anti-4Rtau antibodies showed a similar band pattern from those seen with the anti-P-tauSer422 antibody. In contrast, only weak bands of about 55 kDa, 50 kDa, and 37 kDa were identified with anti-3Rtau antibodies, even used at a concentration greater than that used for immunohistochemistry. Importantly, no bands of 64 kDa were obtained with this antibody even with long exposure times. Smears of molecular weight higher than 75 kDa were noted with anti-P-tauSer422 and anti-4Rtau antibodies (Fig. 9). Western blots of sarkosyl-insoluble fractions of the frontal cortex from GGT linked to *MAPT* P301T mutation, cases 3 and 4 stained with anti-P-tauSer422 antibodies, showed two bands of 68 kDa and 64 kDa, a doublet of about 50 kDa, weak bands or smears of about 40 kDa, one band of about 35 kDa, and a faint, almost absent smear of about 25 kDa (Fig. 9).

Sarkosyl-insoluble fractions of frontal cortex homogenates from sGGT case blotted with anti-P-tauSer422 antibodies showed a band pattern very similar to cases 3 and 4, but with more precise bands of about 50 kDa and 35 kDa, and one lower band of about 20 kDa (Fig. 9).

Western blots of sarkosyl-insoluble fractions of the frontal cortex from GGT linked to *MAPT* K317M mutation showed two bands of 68 kDa and 64 kDa, a smear of about 60 kDa, smears of about 40 kDa, and, lower down, a weak band of about 25 kDa (Fig. 9).

Finally, western blots of sarkosyl-insoluble fractions of the frontal cortex from one case of AD stage VI of Braak and Braak used as a positive control showed bands of 68 kDa, 64 kDa, and 60 kDa, lower bands of about 40 kDa and 35 kDa, and a still lower band of about 25 kDa (Fig. 9).

### Mice inoculated with sarkosyl-insoluble and soluble fractions from GGT linked to *MAPT* P301T mutation

**Fig. 10:**
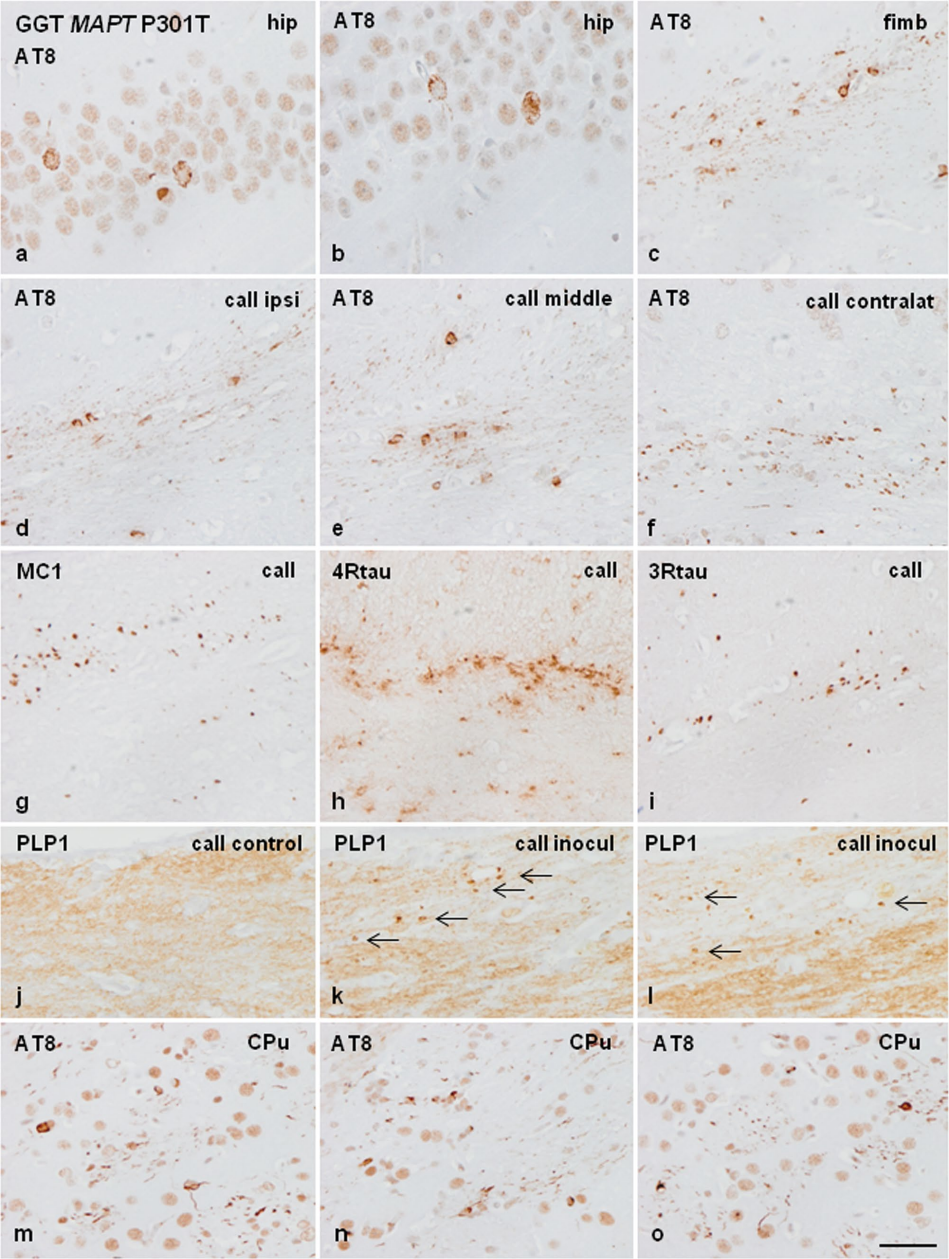
Phospho-tau deposits in neurons of the hippocampus (**a, b**) and in glial cells, threads, and dots in the fimbria (**c**) following unilateral inoculation of sarkosyl-insoluble fractions from the frontal cortex of GGT linked to *MAPT* P301T mutation into the right hippocampus of WT mice aged 12 months and killed 6 months later. Phospho-tau deposits are also seen in glial cells, threads, and dots in the ipsilateral (**d**), middle region (**e**), and contralateral corpus callosum (**f**) in mice inoculated at the age of 12 months and killed at the age of 18 months. Deposits are immunostained with antibodies MC-1 and anti-4Rtau (**g**, **h**). Some deposits are also identified with antibodies against 3Rtau (**i**). **j**–**l** PLP1 immunohistochemistry reveals reduced intensity and the presence of small PLP1-immunoreactive dots (arrows) in the corpus callosum in mice after 4 and 7 months following unilateral inoculation of sarkosyl-insoluble fractions from GGT linked to *MAPT* P301T mutation when compared with controls. **m**–**o** Phospho-tau deposits in the CPu following unilateral inoculation in the right caudate/putamen in mice aged 10 months and killed 5 months later; deposits are found in neurons, glial cells, and numerous threads in the stripes. *hip* hippocampus, *fimb* fimbria, *callipsi* middle, *contralat* corpus callosum ipsilateral, middle region and contralateral, *call* corpus callosum, *CPu* caudate/putamen. Paraffin sections processed for immunohistochemistry slightly counterstained with haematoxylin, bar = 50 μm

Mice inoculated in the right hippocampus with sarkosyl-insoluble fractions from the frontal cortex of GGT linked to *MAPT* P301T mutation case 1 at the age of 12 months and killed at the age of 18–19 months (*n* = 4) showed phospho-tau-immunoreactive cells, as revealed with the AT8 antibody, in ipsilateral neurons of the CA1 region of the hippocampus and dentate gyrus, and in glial cells, dots and threads in the fimbria and corpus callosum (Fig. 10a–c).

Three mice inoculated in the right hippocampus with sarkosyl-insoluble fractions from the white matter of the same GGT case at the age of 7 months and killed at the age of 14 months showed similar deposits to those observed following inoculation of frontal cortex homogenates. However, no deposits were identified in one mouse (data not shown).

Mice inoculated in the right corpus callosum at the age of 7 months and killed at the age of 11 months (survival 4 months) showed phospho-tau-immunoreactive deposits along the ipsilateral and middle region of corpus callosum in glial cells, threads, and dots. Following inoculation into the right corpus callosum in three mice aged 12 months and killed 6–7 months later, large numbers of glial cells, threads, and dots were identified in the ipsilateral, middle region, and contralateral corpus callosum with antibodies AT8 and anti-4Rtau. Dots and threads were also stained with anti-3Rtau and MC-1 antibodies. Tau-C3 and tau-22 immunostaining was negative (Fig. 10d–i). Ubiquitin and p62 were negative except for a few ubiquitin dots in the proximal corpus callosum. One mouse inoculated in the corpus callosum did not show tau deposits.

PLP1 immunohistochemistry revealed decreased immunoreactivity and the presence of small PLP1-immunoreactive dots in the ipsilateral corpus callosum at 4 and 7 months following inoculation of GGT sarkosyl-insoluble fractions into the corpus callosum (Fig. 10j–l).

Finally, inoculation of GGT sarkosyl-insoluble fractions into the right caudate/putamen (CPu) in mice aged 10 months and killed five months later showed phospho-tau deposits, as revealed with AT8 antibody in neurons and glial cells, and in threads in the local fibre bundles which form the stripes (Fig. 10m–o). In contrast to injection into the hippocampus and corpus callosum, inoculation into the right CPu produced more restricted spreading. Phospho-tau immunoreactivity was present only in the vicinity of the site of injection. A few immunoreactive fibres were seen in the internal capsule and in the corpus callosum, probably related to the diffusion of the inoculum at the time of injection.

Apoptosis, as assessed with the method of in situ end-labelling of nuclear DNA fragmentation, was not seen in inoculated mice at the time-points examined in the present study. Reactive astrogliosis and microgliosis, as revealed with anti-GFAP and Iba1 antibodies, respectively, were absent.

Mice inoculated with sarkosyl-soluble fractions and the mouse inoculated with vehicle alone did not show phospho-tau-immunoreactive deposits (data not shown).

**Fig. 11:**
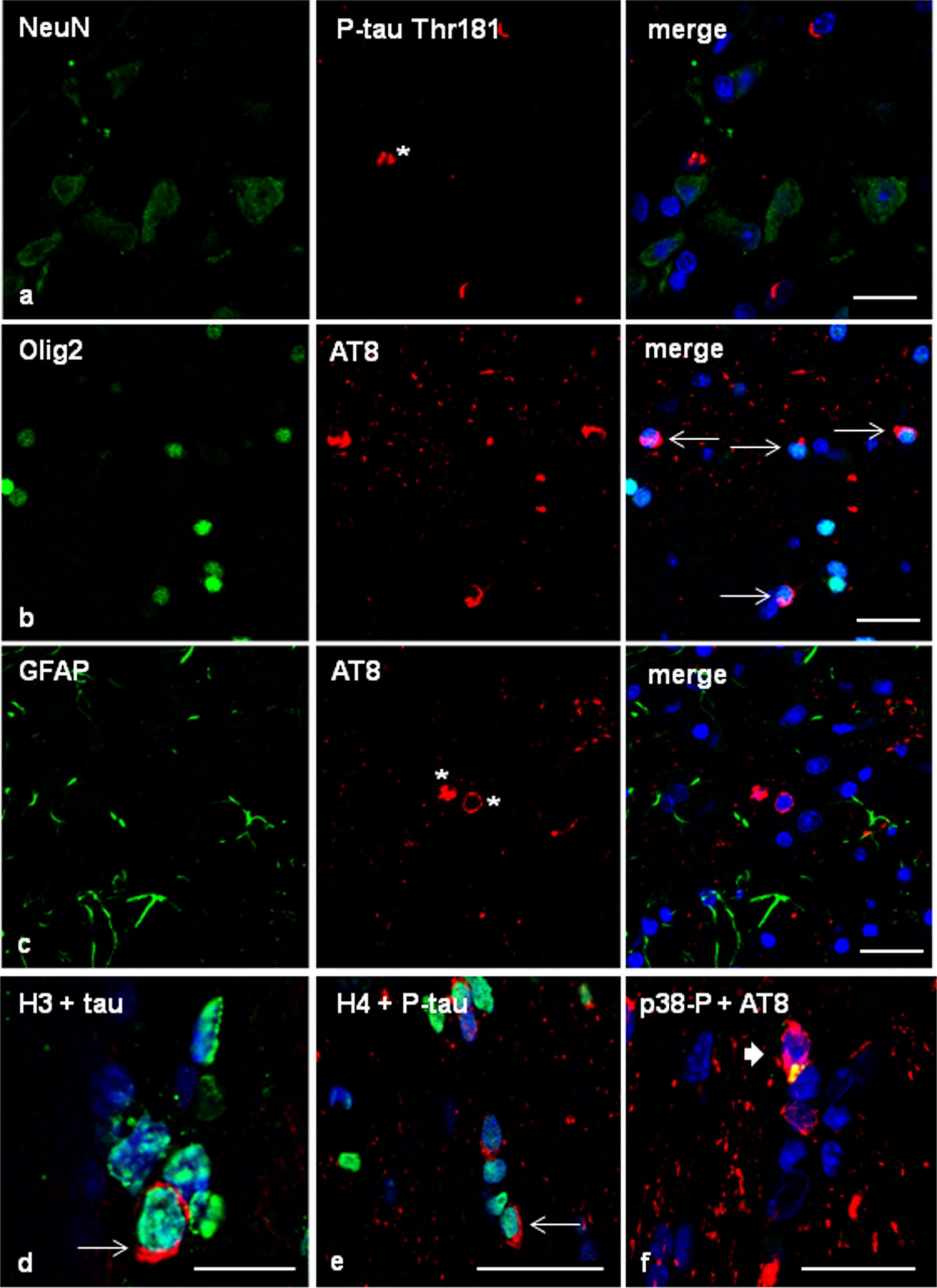
**a–c** Double-labelling immunofluorescence and confocal microscopy to NeuN, Olig2, and GFAP, and phospho-tau specific antibodies P-tauThr181, and AT8, respectively, in the fimbria (**a**) and corpus callosum (**b**, **c**) of WT mice injected with sarkosyl-insoluble fractions from GGT linked to *MAPT* P301T mutation into the hippocampus at the age of 12 months and killed at the age of 18 months. Phospho-tau inclusions are observed only in oligodendrocytes (arrows). Tau deposits do not co-localize with neuronal and astrocytic markers (asterisk). **d** Double-labelling immunofluorescence to H3K9me2 and P-tauThr181, and **e** double-labelling to H4K12ac and phospho-tau AT8 in the corpus callosum of WT mice inoculated with sarkosyl-insoluble fractions of GGT linked to *MAPT* P301T mutation, showing histone modifications expressed equally in cells with (thin arrows) and without hyper-phosphorylated tau deposits (**d**, **e**). Histone H4 (acetyl K12) immunoreactivity is expressed equally in oligodendrocytes with coiled bodies (thin arrows) and oligodendroglia without tau inclusions. **f** Phosphorylated p38 Thr180-Tyr182 (p38P) co-localizes with AT8-immunoreactive deposits (asterisk) in glial cells in the corpus callosum of WT mice inoculated with sarkosyl-insoluble fractions of GGT at the age of 12 months and killed at the age 18 of months. Nuclei are counterstained with DRAQ5™ (blue). Paraffin sections, **a**, **b**, and **c** bars = 20 μm, 24 μm and 30 μm; **d**, **e**, and **f** bars = 10 μm, 30 μm, and 20 μm, respectively

The type of phospho-tau-immunolabelled cells following inoculation of sarkosyl-insoluble fractions was recognized by double-labelling immunofluorescence and confocal microscopy with anti-tau antibodies and specific neuronal (NeuN), astroglial (GFAP), oligodendroglial (Olig2), and microglial (Iba1) antibody markers. This procedure identified NeuN and P-tauThr181 co-localization in neurons in the hippocampus in mice subjected to inoculation in the hippocampus (data not shown). Oligodendroglial cells, as revealed with the Olig2 and AT8 antibodies, were the only population of tau-containing glial cells in the fimbria and corpus callosum at 6–7 months following inoculation of sarkosyl-insoluble fractions in the hippocampus and corpus callosum (Fig. 11a–c). Quantitative studies were carried out in mice inoculated in the corpus callosum with sarkosyl-insoluble fractions and killed at 6–7 months after injection, in sections processed for double-labelling immunofluorescence and confocal microscopy. Counts revealed that about 30% of the total number of oligodendrocytes in the ipsilateral corpus callosum contained phospho-tau deposits in mice inoculated with GGT homogenates. In no case did phospho-tau deposits have the appearance of GOIs as seen in human cases. Rather, oligodendroglial inclusions had coma-like (coiled bodies) or perinuclear circular morphology. Double-labelling immunofluorescence and confocal microscopy disclosed no differences in the expression of H3K12ac and H4K12ac in glial cells with and without phospho-tau deposits following inoculation into the corpus callosum (Fig. 11d, e). Finally, double-labelling immunofluorescence with phosphorylated-p38 and AT8 antibodies revealed co-localization in about 20% of glial cells containing phospho-tau in the corpus callosum (Fig. 11f).

Gallyas staining showed negative or, very rarely, faint positive neurons in the hippocampus, and Gallyas-positive coiled bodies in the white matter (Supplementary Fig. 5).

### Mice inoculated with sarkosyl-insoluble and soluble fractions from sporadic GGT and familial GGT linked to *MAPT* K317M mutation

**Fig. 12:**
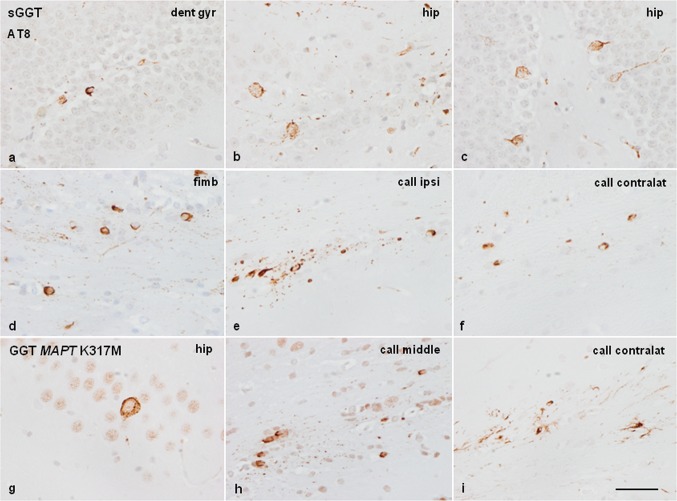
**a**–**f** WT mice inoculated unilaterally into the right hippocampus and corpus callosum with sarkosyl-insoluble fractions from sGGT at the age of 3 months and killed at the age of 9 months. Phosphorylated tau deposits are seen in neurons of the hippocampus (**b**, **c**), glial cells in the hilus of the dentate gyrus (**a**); variegated cells, threads and dots in the fimbria (**d**); and glial cells, threads and dots in the ipsilateral (**e**) and contralateral (**f)** corpus callosum. **g**–**i** WT mice inoculated into the right hippocampus and corpus callosum with sarkosyl-insoluble fractions from GGT linked to *MAPT* K317M mutation at the age of 4 months and killed at the age of 10 months. Phosphorylated tau deposits are seen in one neuron of the hippocampus (**g**) and in glial cells, threads and dots in the middle region (**h**), and contralateral (**i)** corpus callosum. Tau deposits in neurons are characterized by small cytoplasmic granules often expanding to the apical dendrites. Tau deposits in glial cells are perinuclear, often elongated or forming coiled bodies. GGIs are not observed. *hip* hippocampus, *fimb* fimbria, *dentgyr* dentate gyrus, *callipsi* middle, *contralat* corpus callosum ipsilateral, middle region, and contralateral. Paraffin sections processed for immunohistochemistry slightly counterstained with haematoxylin, bar = 50 μm

WT mice inoculated in the right hippocampus and corpus callosum with sarkosyl-insoluble fractions from sGGT and GGT linked to *MAPT* K317M mutation showed the same profile as those inoculated with sarkosyl-insoluble fractions from GGT cases linked to *MAPT* P301T mutation. Mice inoculated in the hippocampus at the age of 3–4 months and killed 6 months later showed phospho-tau deposits in neurons of the hippocampus and dentate gyrus, and glial cells in the hilus and fimbria, together with glial cells, threads, and dots in the ipsilateral, middle region, and contralateral corpus callosum. Tau deposits in neurons were characterized by small cytoplasmic granules often extending to the apical dendrites. Phospho-tau deposits in glial cells were perinuclear, often elongated or forming coiled bodies (Fig. 12). GGIs were not observed. Double-labelling immunofluorescence using specific glial cell markers identified oligodendroglial cells as the only glia containing phospho-tau deposits.

Neurons were Gallyas-negative and coiled bodies Gallyas-positive. Further details were recognized using double-labelling immunofluorescence and confocal microscopy in mice inoculated with sarkosyl-insoluble fractions from GGT linked to *MAPT* K317M mutation. Most oligodendroglial inclusions were coiled bodies, but a minority showed bizarre cytoplasmic tau-positive inclusions and, very rarely, a tau-positive globular-like appendix in the cytoplasm (Supplementary Fig. 7).

### Comparison of tau deposits in human GGT and GGT-inoculated WT mice

The main aspects were the following: (1) neuronal tau inclusions in GGT cases were tangles and pretangles, whereas phospho-tau deposits in inoculated mice had the characteristics of pretangles; (2) tau deposits in GGT cases were phosphorylated, had abnormal conformation and truncation at aspartic acid 421, contained oligomeric tau, and were overwhelmingly composed of 4Rtau. In contrast, phospho-tau deposits in inoculated mice did not contain truncated tau and oligomeric tau and were composed of 4Rtau and 3Rtau; (3) phospho-tau deposits in GGT involved neurons, astrocytes (GAIs), oligodendrocytes (GOIs and coiled bodies), and threads; tau inoculation in mice involved neurons and oligodendrocytes (coiled bodies) in addition to threads; GOIs and GAIs were not observed in inoculated mice; (4) similar deposits were found in inoculated mice using sarkosyl-insoluble fractions from GGT linked to *MAPT* P301T mutations, GGT linked to *MAPT* K317M mutation, and sGGT, excepting minor particularities in GGT linked to *MAPT* K317M mutation; (5) similar characteristics regarding Gallyas staining were seen in GGT cases and inoculated mice; and (6) GGT cases had serious damage in the grey matter and white matter, a feature which was not reproduced in inoculated mice following injection in the hippocampus, caudate/putamen, and corpus callosum, excepting loss of myelin in the ipsilateral corpus callosum.

## Discussion

The present observations are focused on familial GGT linked to *MAPT* P301T mutation with additional studies on sporadic GGT and GGT linked to *MAPT* K317M mutation. Clinical and neuropathological alterations in the familial GGT cases linked to *MAPT* P301T mutations are similar to those reported in sporadic GGT cases [1]. The clinical manifestations and the distribution and localization of pathological changes in the present series differ from one case to another among patients bearing the same mutation and even among affected members of the same family, as previously reported in other tauopathies linked to *MAPT* mutations [10, 28, 38, 42, 47, 112, 135]. GGT linked to *MAPT* P301T mutation cases 2, 3, and 4 is tentatively categorized as subtype II, case 1 as subtype III; the case of sGGT is classified as subtype I, and the GGT case linked to *MAPT* K317M mutation to subtype III, following the nomenclature proposed in sporadic GGT [1]. However, in addition to the basic traits of the three subtypes, several morphological particularities of the inclusions are distinguished in the different cases. Regarding neurons, globular Pick-like inclusions have been considered typical of subtype III in sporadic GGT [116], but here similar inclusions are observed in cases 2, 3, and 4, classified as subtype II. Regarding astrocytes, GAIs with globular perikaryal inclusions are most common in subtype III and GAIs with large radiating process-like structures are more common in subtype II [116]. This also applies in our series. Yet GAIs with radiating processes are also seen in case 1 with *MAPT* P301T mutation and in the case with *MAPT* K317M mutation. Immature forms are present in all GGT cases in consonance with earlier observations [116]. Finally, astrocyte-like plaques occur in familial cases categorized as subtype III.

Biochemical profiles also differ from one case to another in the present series, independently of the age at onset, gender and duration of the disease. The basic two-band pattern, as revealed by western blotting with anti-P-tauSer422 antibodies, of 68 kDa and 64 kDa characteristic of 4Rtau tauopathies, already reported in other cases suffering from GGT [3, 7, 28, 38, 45, 111, 135], is regular in all cases. However, regarding tau species, the band patterns of phosphorylated tau differ from one case to another in the same cortical region (frontal cortex area 8) even in cases carrying the same mutation. The profile of case 1 linked to *MAPT* P301T mutation is subtly alike the profiles of cases 3 and 4 carrying the same mutation. This is manifested in the intensity of bands of about 50 kDa which are better defined in cases 3 and 4, and the bands of about 40 kDa which are stronger in case 1. The bands of truncated phospho-tau at carboxy terminal (bands of about 25 and 20 kDa) are almost absent in cases 3 and 4. This last characteristic is consistent with the presence of  Tau-C3 (which recognizes tau truncated at aspartic acid 421) in case 1 and the almost total absence of tau-C3 with immunohistochemistry in cases 3 and 4.

The band pattern in sGGT is similar to that seen in cases 3 and 4 bearing the *MAPT* P301T mutation; in accordance, Tau-C3 immunoreactivity is restricted to a subpopulation of oligodendroglial inclusions. The study of bands by western blotting in GGT linked to *MAPT* K317M mutation is suboptimal due to the poor preservation of the sample, as suggested by the appearance of smears below the two bands of 68 kDa and 64 kDa, particularly below 50 kDa. Yet the band of about 25 kDa is visible in agreement with the presence of Tau-C3-immunoreactive inclusions in tissue sections. Oligomeric bands are more marked in case 1 when compared with the other cases.

Finally, differences between the frontal cortex and immediate subcortical white matter are also seen, at least in case 1, the only one assessed. The intensity of bands is lower in the white matter excepting one band below 37 kDa, the intensity of which is lower in the cerebral cortex.

The biochemical profile of phospho-tau in GGT cases differs from that seen in corticobasal degeneration (CBD) and progressive supranuclear palsy (PSP). On immunoblots of sarkosyl-insoluble brain extracts, a 33 kDa band predominates in the low molecular weight tau fragments in PSP, whereas two closely related bands of approximately 37 kDa predominate in CBD [5, 117]. The patterns described in PSP and CBD appear to be identical from one patient to another. However, our observations in GGT show, in addition, certain individual variability in GGT. Considering the clinical and neuropathological variability of PSP, Richardson’s syndrome, and CBD, whether individual variabilities occur in CBD and PSP deserves further study.

The site of mutation in the pathogenesis of familial GGT does not shed light on the particular phenotype in familial cases, as mutations causative of familial GGT are linked to exon 10, exon 11, exon 1, and to IVS10 + 16 [10, 44, 56, 62, 111, 112, 135]. The small number of reported cases of familial GGT makes it difficult to ascribe individual changes to the different mutations in *MAPT,* as suggested by some researchers [110, 111]. It is more likely the case that familial GGT, like sporadic GGT, is subjected to currently unknown biochemical factors which demarcate individual-specific phenotypes independently of the genetic defect. Based on newly available information, the classification of GGT into different subtypes proposed a few years ago [1] should be interpreted with caution and viewed as an instrumental categorization subjected to refinement [11]. Further studies are needed to better understand cellular and regional vulnerability in frontotemporal tauopathies [43].

### Astrocytopathy and oligodendrocytopathy in GGT

Most neurodegenerative diseases with abnormal protein aggregates can no longer be viewed solely as neuronopathies. Abnormalities in astrocytes and oligodendrocytes imply key roles for glial cells in distinct neurological diseases. Astrocytopathy and oligodendrogliopathy have emerged as main components of neurodegenerative diseases with abnormal protein aggregates [8, 29, 30, 33–35, 60, 86, 94, 97, 99, 103, 123–125, 129, 132].

Alterations of astrocytes and oligodendrocytes in GGT are barely known except for the description of characteristic morphology of GAIs and GOIs, including Gallyas-negative GAIs, and Gallyas-positive GOIs and coiled bodies [33, 34, 67]. For this reason, the levels of gene transcripts of astrocytes and oligodendrocytes are assessed separately in the frontal cortex and subcortical frontal white matter in familial GGT carrying the *MAPT* P301T mutation. Despite the limitations inherent in the small number of pathological samples, mRNA expression of basal, reactive, and inflammatory markers of astrocytes (*ALDH1L1*, *GFAP*, *YKL40*, respectively) is increased in the frontal cortex in GGT. *GJA1* coding for gap junction α1-protein/connexin-43, the major constituent of gap junctions in astrocytes, is also significantly increased, thus supporting the idea of an extended astrocytic network in GGT which would facilitate, at least in normal conditions, the transfer of small molecules and metabolites such as glucose and lactate [48, 52, 91]. Whether those gap-mediated functions are at work in GGT is not known.

The present findings show reactive astrocytosis characterized by increased GFAP and YKL40. Increased chaperone responses manifested as increased αB-crystallin expression in glial cells are also independent of the intracytoplasmic tau inclusions, as already reported in astrocytes in other tauopathies [21, 85]. Yet GFAP is redistributed in the cytoplasm of astrocytes containing tau, a feature also found in other tauopathies with tau deposits in astrocytes [33, 37]. Astrocytopathy in GGT linked to *MAPT* P301T mutation is also manifested by increased AQ4 expression, decreased GLT1, decreased GLUC-t, and altered MPC1 and UCP5, arguing for impaired modulation of water, glutamate, and glucose transport, and altered mitochondrial coupling, in affected astrocytes.

Immunohistochemistry in case 1 discloses that several mRNA alterations are in the same line as those of the expression of corresponding proteins (for example, increased expression of GFAP and YKL40 proteins), and it identifies additional protein alterations in astrocytes in GGT not detected at the mRNA level by RT-qPCR. Some of these alterations are particularly informative by alerting to abnormal function of GGT astrocytes. Aquaporin 4 (AQ4) immunoreactivity is markedly increased in the frontal cortex, suggesting abnormalities in water transport between astrocytes and blood vessels. Alterations in AQ4 expression have been reported in age-related tau astrogliopathy (ARTAG) [37, 71], in astrocytes surrounding β-amyloid plaques, and in perivascular astrocytes in AD [60]; reactive astrocytes in bovine spongiform encephalopathy and related models, in which reactive astrocytes do not contain hyper-phosphorylated tau and PrP^SC^, respectively, also express high levels of AQ4 [18]. Similarly, altered expression of astrocytic and oligodendroglial connexins is common in many neurodegenerative diseases independently of the presence of abnormal protein deposits in reactive cells [19, 89, 90, 126]. Therefore, altered AQ4 expression and increased connexin-43 immunoreactivity cannot be considered markers of a particular disease.

Glutamate transporter 1 immunoreactivity is markedly reduced, in spite of the larger number of astrocytes, in GGT linked to *MAPT* P301T mutation, thus suggesting impaired glutamate transport in this disease, leading in turn to increase glutamate excitotoxicity and neuron damage [101]. Abnormal glutamate transport is well documented in a limited number of neurological diseases, most specifically in amyotrophic lateral sclerosis. Abnormal glutamate transport function has also been reported in mutant amyloid precursor protein transgenic mice [87], and altered ratio between GFAP and GLT1 in the cerebral cortex in AD [107]. However, we were unable to find differences in the total expression levels of GLT1 in AD and in dementia with Lewy bodies using the same methods as those used in the present study [46]. GLT1 (EAAT2) expression is markedly reduced in most astrocytes bearing hyper-phosphorylated tau in a rare familial behavioural variant of frontotemporal dementia associated with astrocyte-predominant tauopathy not linked to mutations in *MAPT* [39]. Reduced GLT1 expression also occurs in transgenic mice selectively expressing abnormal hyper-phosphorylated tau in astrocytes [20]. Therefore, abnormal GLT1 expression appears to be a major defect in GGT linked to *MAPT* P301T mutation.

The expression of proteins involved in glucose transport is also abnormal in GGT linked to *MAPT* P301T mutation: GLUC-t encoded by the solute carrier family 2, member 1 (*SLC2A1*) is reduced in the neuropil. Mitochondrial pyruvate carrier 1 (MPC1) immunoreactivity is decreased in neurons in the frontal cortex (in line with results of mRNA expression), but MPC1 is markedly increased in astrocytes in the subcortical white matter (in contrast to mRNA expression levels in the same region). Double-labelling immunofluorescence and confocal microscopy disclose no differences in MPC1 and UCP4 immunoreactivity in cells with and without tau deposits, indicating that decreased MPC1 immunoreactivity in frontal cortex is not linked to coexisting deposition of hyper-phosphorylated tau in the same cells. In contrast, UCP5 immunoreactivity is decreased only in cells containing phospho-tau aggregates when compared with similar cells without phospho-tau deposits in the same tissue sections.

Regarding white matter, *OLIG1* and *OLIG2* mRNA expression levels are significantly reduced in GGT cases linked to *MAPT* P301T mutation compared to controls. This is accompanied by a significant decrease in the expression of all assessed genes involved in myelin formation, including *MBP*, *PLP1*, *CNP*, *MAG*, *MAL*, *MOG,* and *MOBP*. Immunohistochemistry further demonstrates the reduction of CNPase, MBP, and PLP-1 proteins in the white matter in GGT. The mRNA expression of *SLC2A1*, coding for solute carrier family 2 (facilitated glucose transporter), member 1, and *MCT1* coding for the solute carrier family 16 (monocarboxylic acid transporter), member 1 is significantly reduced in GGT when compared with controls. White matter abnormalities have been described in transgenic mice bearing human tau mutations in which neurons, astrocytes, and oligodendrocytes accumulate filamentous inclusions [80, 81]. More telling is the observation that selective over-expression of mutant tau in oligodendrocytes using *CNP* promoter in mice produces filamentous inclusions in oligodendrocytes, and progressive impairment of axonal transport followed by myelin and axonal disruption [58].

In light of the evidence, we can consider that, in addition to impaired white matter connectivity due to tau deposits in neurons and threads, oligodendrocytopathy is a key factor in the pathogenesis of GGT linked to *MAPT* P301T mutation, not only because of the presence of phospho-tau deposits conforming GOIs and coiled bodies, but more importantly because these deposits have consequences in oligodendrocyte function, leading to defective myelin synthesis, demyelination, oligodendroglial energy metabolism dysfunction, and concomitant axonal damage manifested as disrupted axons, and axonal varicosities and spheroids filled with phosphorylated H neurofilaments.

Only the expression of a few proteins was analysed in the frontal cortex in sGGT and in GGT linked to *MAPT* K317M mutation due to the limited availability of tissue. YKL40 immunoreactivity is markedly increased in GGT linked to *MAPT* K317M mutation. GLUC-t and GLT1 immunoreactivity is markedly reduced in the neuropil in sGGT and GGT linked to *MAPT* K317M mutation. These aspects are similar to those seen in the cases of GGT linked to *MAPT* P301T mutation. Regarding AQ4, it is preserved around blood vessels in sGGT and reduced in frontal cortex in *MAPT* linked to *MAPT* K317M mutation. The reason of differences in AQ4 expression between GGT linked to *MAPT* P301T mutation, and sGGT and *MAPT* linked to K317M mutation, cannot be explored in detail because of the small number of cases.

The question of increased numbers of capillaries in the frontal cortex in GGT has not been previously addressed. The number of capillaries in the frontal cortex, as revealed with GLUC-t immunohistochemistry, is significantly increased in all GGT cases when compared with controls in the present series. Our hypothesis is that this effect is due to the neuronal loss and cortical atrophy of frontal degeneration in GGT cases, together with angiogenesis (capillary sprouting) induced by protein pathology and neuroinflammation, as proposed in ageing and other brain diseases [93].

### GGT: not only hyper-phosphorylated tau in neurons and glial cells

In addition to hallmark pathological features specific for every particular neurodegenerative disease, the use of combined “omics” has delineated a more complex profile involving a large number of altered expression profiles of proteins and metabolites, and altered biochemical pathways in the majority of neurodegenerative diseases with abnormal protein aggregates [73–75]. Some of these have been identified at early stages of the disease without apparent connection with the localization of pathogenic protein aggregates [32]. Knowledge of these alterations indicates that the occurrence of protein aggregates is not the only alteration in the brain of affected individuals. Phosphoproteomics has identified hyper-phosphorylation of a large number of unrelated proteins in AD [12, 22, 24, 77, 113, 115, 121, 134] and ARTAG [37]. Based on these observations, we hypothesized that widespread phosphorylation changes, as identified in AD and ARTAG, may occur in other tauopathies such as GGT. Our structure-resolved analysis of the proteomic and phosphoproteomic changes that occur in familial GGT linked to *MAPT* P301T mutation reveals that the perturbed proteostasis is more evident in frontal cortex than in white matter. Functional annotation shows that 55% of differential phosphosites detected in frontal cortex are located on proteins involved in neuron projection morphogenesis and chemical synaptic transmission, revealing novel phosphorylation-dependent regulatory paradigms for GGT. On the other hand, the deregulation of NEFL, NEFH and INA protein levels as well as the alteration in the phosphorylation state of tau, MAP1A/MAP1B commonly observed in frontal cortex and white matter reinforces the disruption of the neuronal cytoskeleton in GGT. Based on the alteration of the steady- or phosphorylation states of predicted and verified tau interactors such as casein kinase 2 alpha 1, O-linked *N*-acetylglucosamine (GlcNAc) transferase, S100 calcium binding protein B, Rho/Rac guanine nucleotide exchange factor 2, ubiquitin C, and microtubule-associated protein 2 (CSNK2A1, OGT, S100B, ARHGEF2, UBC, and MAP2, respectively) (Supplementary Table 6; Supplementary Fig. 4), our data point up potential fluctuations of the tau interactome in both brain areas, yielding new insights into the pathophysiological role of tau in GGT. Although our study uncovers many intricacies in frontal cortex and white matter molecular homeostasis in GGT, there are potential limitations to our prototyping study that warrant discussion. First, due to technological issues, we failed to accurately quantify many (phospho)proteins expressed at low levels that might also participate in the GGT phenotype. Second, although our mass-spectrometry approach revealed changes in the phosphorylation profile of tau protein in both regions, the detected phosphorylated sequences are common to the six tau isoforms expressed in human brain, hampering unequivocal assignment. Third, our results are limited by protein abundance averaging among multiple cell layers, hampering the exploration of cell-type specific molecular alterations.

Together, these observations reveal GGT to be linked to *MAPT* P301T mutation as a disorder of protein phosphorylation affecting different substrates and not only protein tau.

### Seeding and spreading of GGT tau following intra-cerebral inoculation in WT mice

Phospho-tau seeding and spreading occurs following inoculation of preformed synthetic tau fibrils [61], and injection of fibrillar-enriched fractions from human or mouse brain homogenates of tauopathies, including AD, only tangle dementia, argyrophilic grain disease, PSP, and CBD, injected into the brain of transgenic mice expressing 4R human tau or human mutant tau [2, 9, 14–16]. Abnormal tau deposits are generated in neurons related to connectivity rather than to proximity [2]. Deposits in glial cells appear to have disease-dependent morphology in inoculated transgenic mice expressing 4R human tau or mutant human tau [9, 14–16].

Similar inoculations carried out in WT mice have shown few neuronal, oligodendroglial, or thread inclusions regardless of the origin of the inclusions [14]. Other studies have reported only neurons and threads as targets of tau seeding following inoculation of AD homogenates, but neurons and glial cells, reminiscent of astrocytic plaques and tufted astrocytes, following inoculation of tau obtained from CBD and PSP brain homogenates, respectively [54, 92]. Another set of experiments described grains and threads but not deposits in neurons and glial cells following inoculation of paired helical filaments from AD cases in the brain of WT mice [6]. Our previous studies have shown a rather homogeneous pattern of tau seeding and spreading following inoculation of sarkosyl-insoluble fractions into the hippocampus or into the corpus callosum of WT mice, independently of the tauopathy. Nerve fibres and coiled bodies were the only tau deposits in the corpus callosum following inoculation of homogenates from AD, primary age-related tauopathy (PART), ARTAG, GGT, PSP, Pick’s disease (PiD), and frontotemporal lobar degeneration linked to *MAPT* P301L mutation (FTLD-P301L) [36]. Neurons and oligodendrocytes were the main targets following inoculation of sarkosyl-insoluble fractions of AD, PART, and ARTAG into the hippocampus of WT mice [37, 41]. No GAIs, GOIs, or thorn-shaped astrocytes (characteristic of ARTAG) were seen following inoculation of GGT and ARTAG, respectively.

The present findings following unilateral inoculation of sarkosyl-insoluble fractions of GGT into the hippocampus, together with the observations of previous studies, are consistent with the idea that tau can be transmitted trans-synaptically from one neuron to another neuron, and that this mechanism has implications in the progression of abnormal tau deposits in tauopathies [27, 49, 50, 79, 83, 106]. Seeding and spreading along neuronal connections following intracellular injection of tau synthetic preformed fibrils is accompanied by altered synaptic transmission, motor deficits, and abnormal behaviour [109].

Trans-synaptic transmission is certainly not the mechanism leading to the spreading of phospho-tau in oligodendrocytes. Since phospho-tau-containing oligodendrocytes are associated with abnormal tau accumulation along tracts, it may be inferred that tau-containing oligodendrocytes are bystanders linked to phospho-tau deposition in axons. However, phospho-tau deposition in oligodendrocytes may be not merely a passive phenomenon, as active phospho-p38 kinase expression co-localizes in oligodendrocytes containing tau, thus suggesting active tau phosphorylation [31, 98]. Several lines of evidence support this interpretation. Endogenous mouse tau is deposited in oligodendrocytes in mice expressing transgenic human tau, in a way that makes it evident that mouse tau has the capacity to be recruited and aggregated in oligodendrocytes [100]. Similarly, sarkosyl-insoluble fractions containing abnormal tau can recruit endogenous murine tau in the present paradigm using GGT homogenates. The transmission of phospho-tau along oligodendrocytes may be explained by other mechanisms, particularly when referring to long tracts, as in the corpus callosum, where oligodendrocytes are the main cellular population [133], and the only cell type containing phospho-tau in our model. Alternative mechanisms proposed in cellular models include tau release using ectosomes or exosomes, freely released to the extracellular space, and uptake through endocytosis and receptor-mediated endocytosis, tunneling nanotubues, and use of extracellular vesicles as shuttles [23, 26, 50, 51, 53, 59, 76, 79, 118, 127, 128, 130, 131]. Deposition of phospho-tau in oligodendrocytes has consequences, as decreased PLP-1 immunoreactivity and small PLP1-immunoreactive dots appear in the proximal corpus callosum following inoculation of GGT sarkosyl-insoluble fractions, suggesting damage to the white matter.

Epigenetic changes may regulate gene transcription. Previous studies showed that expression of dimethylated histone H3K9 (H3K9me2) and acetylated histone H3K12 (H3K12ac), which regulate rRNA transcription [17, 114], decreased in AD with disease progression [57]. However, no differences in the expression of these histones were seen in GGT cases or in tau-containing neurons and glial cells in inoculated mice with sarkosyl-insoluble fractions of human GGT homogenates. These negative results do not rule out the possibility of epigenetic regulation of active pathways linked to tau phosphorylation and other metabolic alterations linked to tau seeding in recruited target cells.

Finally, it is important to stress that the tau biosensor cell line expressing human tau exposed to sporadic or mutant GGT brain lysates reveals large deposits reminiscent of globular inclusions in GGT [13]. However, no GAIs or GOIs, but only coiled bodies, are regularly seen in WT mice inoculated with sarkosyl-insoluble fractions of GGT linked to *MAPT* P301T mutation, sGGT, and GGT linked to *MAPT* K317M mutation in our study. The exception of a few bizarre oligodendroglial inclusions following inoculation of homogenates from *MAPT* K317M samples does not detract from the general concept that coiled bodies are the characteristic oligodendroglial phospho-tau inclusions in WT mice inoculated with homogenates from GGT and other tauopathies [36, 37, 41]. This suggests that host tau is crucial to recapitulate typical inclusions in human diseases. In this line, murine tau differs from human tau in several aspects including the predominance of 4Rtau in adulthood, the C-terminal domain, the N-terminal domain, binding sites, and distribution and localization of the different tau isoforms [64, 78, 82, 88, 102, 118]. These differences may account for the species’ specificity to tau vulnerability, a feature known as species barrier in prion diseases [55, 104]. As a working hypothesis, regional differences in tau may also participate in selective regional and cellular vulnerability. Therefore, not only tau strains of the donor [25, 122] but also tau strains in the host may be critical to refining the characteristics of abnormal protein deposits in degenerative diseases with abnormal protein aggregates.

## Electronic supplementary material

Below is the link to the electronic supplementary material.

Supplementary file1 Supplementary Figure 1 Representative alterations in GGT linked to *MAPT* P301T mutation, case 3. Marked neuron loss and astrogliosis in the frontal cortex (FC) and moderate loss in the locus ceruleus (LC) (a, b); astrogliosis is visualized with anti-GFAP antibodies as seen in FC and thalamus (Thal) (c, d). Many reactive astrocytes are also immunoreactive with anti-αB-crystallin (αBCryst) antibodies (e). In contrast, microgliosis is discrete as seen with the Iba1 antibody (f). Phospho-tau deposits, as revealed with the AT8 antibody, are seen in several brain regions, including the occipital cortex (OccC), frontal cortex (FC), substantia nigra pars compacta (SN), locus ceruleus (LC), pontine nuclei (pons), and white matter (WM) (g-l). Neuronal deposits are granular or tangle-shaped, whereas predominant glial inclusions are GAIs (g, h, thick black arrows) and GOIs (k, thick white arrows). Phospho-tau-immunoreactive threads are also observed in the grey and white matter. Inclusions are not stained with anti-3Rtau antibodies (m) but they are strongly immunostained with anti-4Rtau antibodies (n). A subpopulation of inclusions is ubiquitinated (o). Paraffin sections stained with haematoxylin and eosin (a, b), or processed for immunohistochemistry and slightly counterstained with haematoxylin (c-o); bar = 25μm (PDF 230 kb)

Supplementary file2 Supplementary Figure 2 a) Dot graphs representing the expression of astrocyte-associated genes in frontal cortex area 8 in the four GGT cases linked to *MAPT *P301T mutation and 10 controls. *GFAP*, *ALDH1L1*, *YKL40*, and *GJA1* are significantly increased, and *MPC1* significantly decreased in GGT cases when compared with controls. Student’s t test, p<0.05, ** p<0.01 and *** p<0.001 (PDF 235 kb)

Supplementary file3 Supplementary Figure 2 b) Dot graphs representing the expression of oligodendrocyte- and myelin-associated genes in frontal cortex area 8 in the four GGT cases linked to *MAPT* P301T mutation and 10 controls. *NG2 *mRNA expression is significantly increased in GGT cases; however, the majority of myelin-linked genes show a trend to decrease in disease cases. Student’s t test, p<0.05, ** p<0.01 and *** p<0.001 (PDF 314 kb)

Supplementary file4 Supplementary Figure 3 a) Dot graphs representing the expression of astrocyte-associated genes in the frontal subcortical white matter in the four GGT cases linked to *MAPT* P301T mutation and 10 controls. *MPC1* mRNA is significantly decreased in GGT. Student’s t test, p<0.05, ** p<0.01 and *** p<0.001 (PDF 227 kb)

Supplementary file5 Supplementary Figure 3 b) Dot graphs representing the expression of oligodendrocyte- and myelin-associated genes in the frontal subcortical white matter in the four GGT cases linked to *MAPT* P301T mutation and 10 controls shows significant reduction of *OLIG1*, *OLIG2*, *MBP*, *PLP1*, *CNP*, *MAG*, *MAL*, *MOG*, *MOBP*, *SLC2A1*, and *MCT1* in GGT cases compared with controls. Student’s t test, p<0.05, ** p<0.01 and *** p<0.001 (PDF 317 kb)

Supplementary file6 Supplementary Figure 4 Protein interactome network for frontal cortex-modulated (phospho)proteome. Network analysis was performed submitting the corresponding protein IDs to the STRING (Search Tool for the Retrieval of Interacting Genes) software (v.10.5) (http://stringdb.org/). Proteins are represented with nodes and the interactions with continuous lines to represent direct interactions (physical), while indirect ones (functional) are presented by interrupted lines. All the edges were supported by at least one reference from the literature or from canonical information stored in the STRING database. To minimize false positives as well as false negatives, only interactions tagged as “high confidence” (> 0.7) in STRING database were considered. K means clustering was applied (PDF 111 kb)

Supplementary file7 Supplementary Figure 5 Gallyas staining of GGT cases (a-s) and inoculated mice with sarkosyl-insoluble fractions from GGT linked to *MAPT* P301T mutation (t, u). Neurons are variably stained in the cerebral cortex showing tangle-like morphology (a), dense granular staining (b and thick black arrow in d), weak and fine granular staining (c), faint difuse staining, or no staining (d). GAIs are negative, but a small subpopulation of astrocytes show faint granular Gallyas-positive staining in the distal region of astrocytic processes or in the cytoplasm of a few number astrocytes (e, f). Very rare astrocytes with perinuclear round Gallyas-positive deposits are seen in GGT linked to *MAPT* K317M mutation (g). Coiled bodies are positive (h-l), and there are GOIs (m-s) in every case. GOIs are also seen in e and f (thin black arrows). Gallyas-positive threads and bizarre oligodendroglial inclusions are also seen in GGT linked to *MAPT* K317M mutation (o, s). Gallyas-positive glial cells of unknown origin are rarely found in GGT linked to *MAPT* K317M mutation (asterisk in a). Mice inoculated with sarkosyl-insoluble fractions of GGT cases very rarely show faint positive neurons (t) and common Gallyas-positive coiled bodies (u). b, c, d e, h, i, m, n: GGT linked to *MAPT* P301T mutation, case 1; f, j, i, p: case 3; k: case 4; q, r: sGGT; g, l, o, s: GGT linked to *MAPT* K317M mutation. t, u: mouse inoculated at the age of 12 months with sarkosyl-insoluble fractions of GGT linked to *MAPT* linked to P301T mutation, case 1 (survival 6 months). Paraffin sections without counterstaining; a-d, bar = 25µm; e-l, bar = 10 µm; m-s, bar = 15 µm; t, s, bar = 20µm (PDF 180 kb)

Supplementary file8 Supplementary Figure 6 a-f: Aquaporin 4 (AQ4) and glucose transporter (GLUC-t) expression in the frontal cortex (FC) in sGGT and corresponding controls (Contr) processed in parallel. AQ4 immunoreactivity is reduced in sGGT when compared with controls, but AQ4 is still preserved around blood vessels (a-d). In control FC, GLUC-t is heavily expressed in capillaries and moderately so in the neuropil. GLUC-t is increased in sGGT because the higher number of capillaries when compared with controls, but GLUC-t immunoreactivity is decreased in the neuropil (e, f). g-l: YKL40, GLUC-t, glutamate transporter 1 (GLT1) and AQ4 in FC in GGT linked to *MAPT* K317M mutation and corresponding controls. YKL40-immunoreactive astrocytes show huge size and bizarre morphology (g) (compare Figure 3a for YKL40 immunoreactivity in the FC in controls). GLUC-t immunohistochemistry shows increased numbers of capillaries and decreased immunoreaction in the neuropil (h). GLT1 (i, j) and AQ4 (k, l) immunoreactivities are reduced in GGT linked to *MAPT* K317M compared with controls processed in parallel. Paraffin sections, slightly counterstained with haematoxylin; a, c, e, f, h, i and j, bar in j =70µm; b, d, g, k and l, bar in l = 35µm (PDF 306 kb)

Supplementary file9 Supplementary Figure 7 Double-labelling immunofluorescence and confocal microscopy to Olig2 (green) and AT8 (red) in the corpus callosum of mice inoculated with sarkosyl-insoluble fractions from GGT linked to *MAPT *K317M mutation shows typical coiled bodies (a and b)) (thin white arrows) and, more rarely, bizarre oligodendroglial tau-positive inclusions (thin white double-arrow) and tau-positive globular-like appendix in the cytoplasm (thick white arrow (b). The nucleus of one oligodendroglial cell is of a huge size (asterisk). Nuclei are counterstained with DRAQ5TM (blue). Paraffin sections, bar = 20 μm (PDF 117 kb)

Supplementary file10 (RAR 955 kb)
