# Supplementary figures and images for: Familial globular glial tauopathy linked to MAPT mutations: molecular neuropathology and seeding capacity of a prototypical mixed neuronal and glial tauopathy
Source: Acta Neuropathol. 2020 Jan 6;139(4):735–71. doi: 10.1007/s00401-019-02122-9 (PMC7096369; doi:10.1007/s00401-019-02122-9)

Supplementary Figure 1

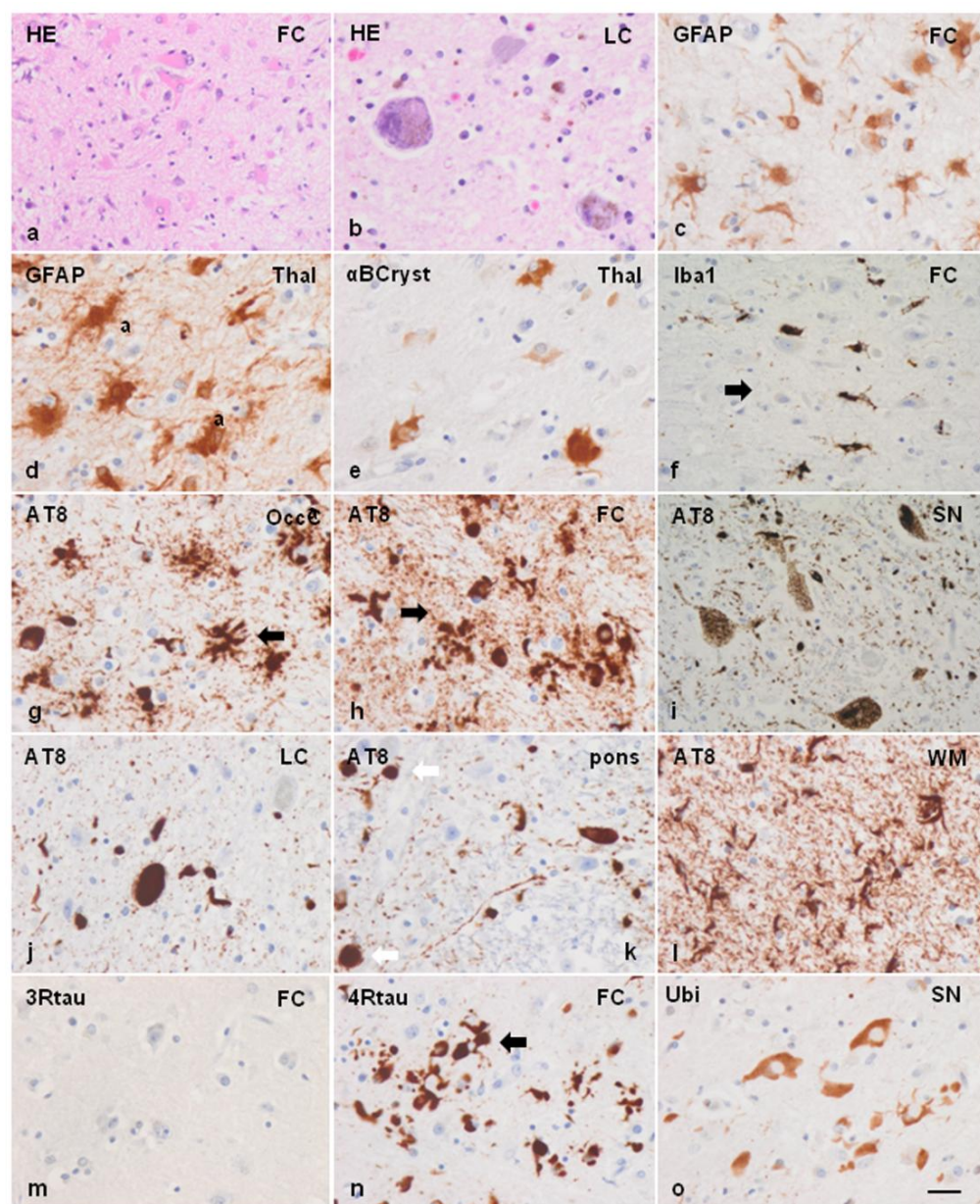

Supplement: Supplementary file 1 — Supplementary file1 Supplementary Figure 1 Representative alterations in GGT linked to MAPT P301T mutation, case 3. Marked neuron loss and astrogliosis in the frontal cortex (FC) and moderate loss in the locus ceruleus (LC) (a, b); astrogliosis is visualized with anti-GFAP antibodies as seen in FC and thalamus (Thal) (c, d). Many reactive astrocytes are also immunoreactive with anti-αB-crystallin (αBCryst) antibodies (e). In contrast, microgliosis is discrete as seen with the Iba1 antibody (f). Phospho-tau deposits, as revealed with the AT8 antibody, are seen in several brain regions, including the occipital cortex (OccC), frontal cortex (FC), substantia nigra pars compacta (SN), locus ceruleus (LC), pontine nuclei (pons), and white matter (WM) (g-l). Neuronal deposits are granular or tangle-shaped, whereas predominant glial inclusions are GAIs (g, h, thick black arrows) and GOIs (k, thick white arrows). Phospho-tau-immunoreactive threads are also observed in the grey and white matter. Inclusions are not stained with anti-3Rtau antibodies (m) but they are strongly immunostained with anti-4Rtau antibodies (n). A subpopulation of inclusions is ubiquitinated (o). Paraffin sections stained with haematoxylin and eosin (a, b), or processed for immunohistochemistry and slightly counterstained with haematoxylin (c-o); bar = 25μm (PDF 230 kb) [file 401_2019_2122_MOESM1_ESM.pdf]

Supplementary Figure 2a

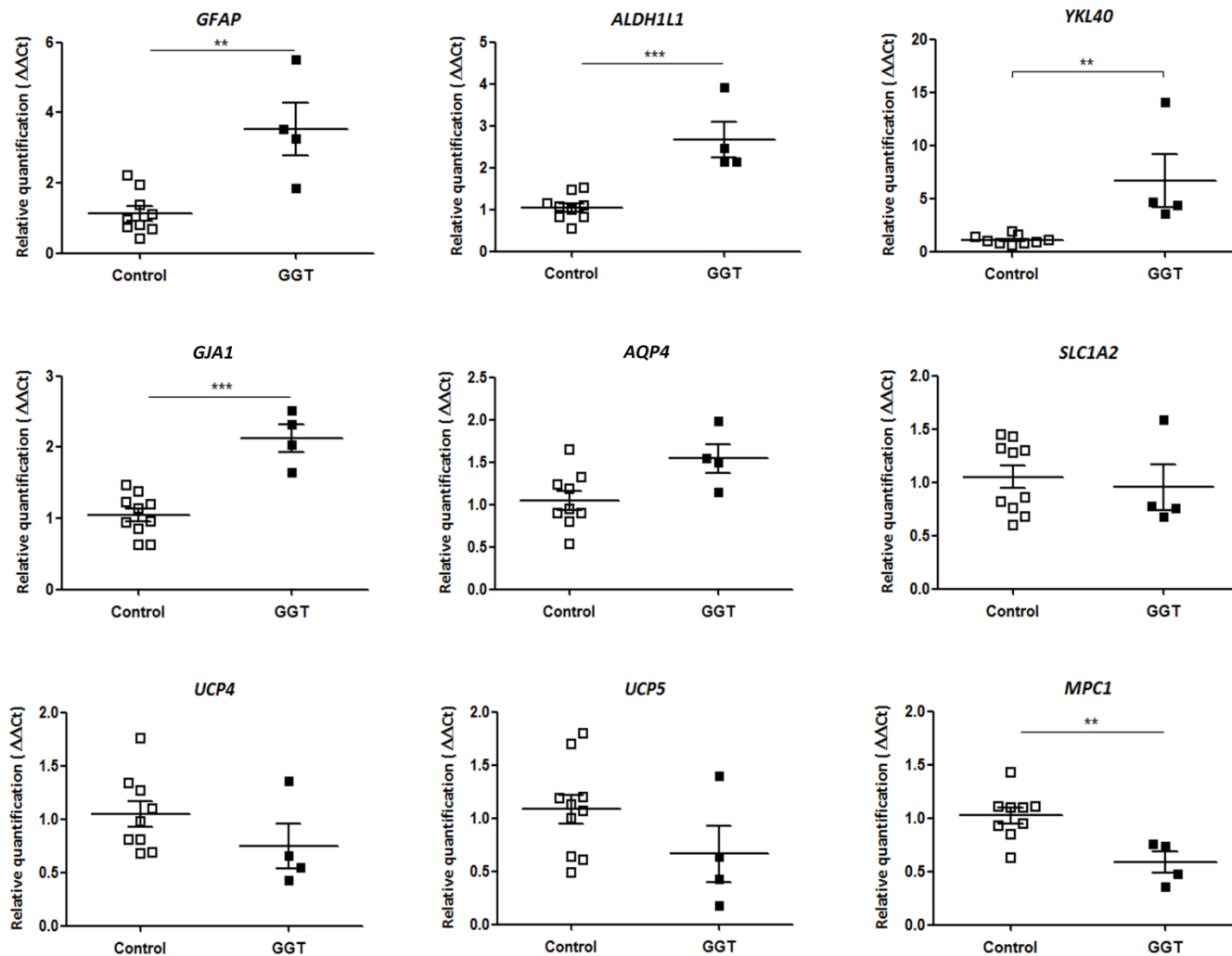

Supplement: Supplementary file 2 — Supplementary file2 Supplementary Figure 2 a) Dot graphs representing the expression of astrocyte-associated genes in frontal cortex area 8 in the four GGT cases linked to MAPT P301T mutation and 10 controls. GFAP, ALDH1L1, YKL40, and GJA1 are significantly increased, and MPC1 significantly decreased in GGT cases when compared with controls. Student’s t test, p<0.05, ** p<0.01 and *** p<0.001 (PDF 235 kb) [file 401_2019_2122_MOESM2_ESM.pdf]

Supplementary Figure 2b

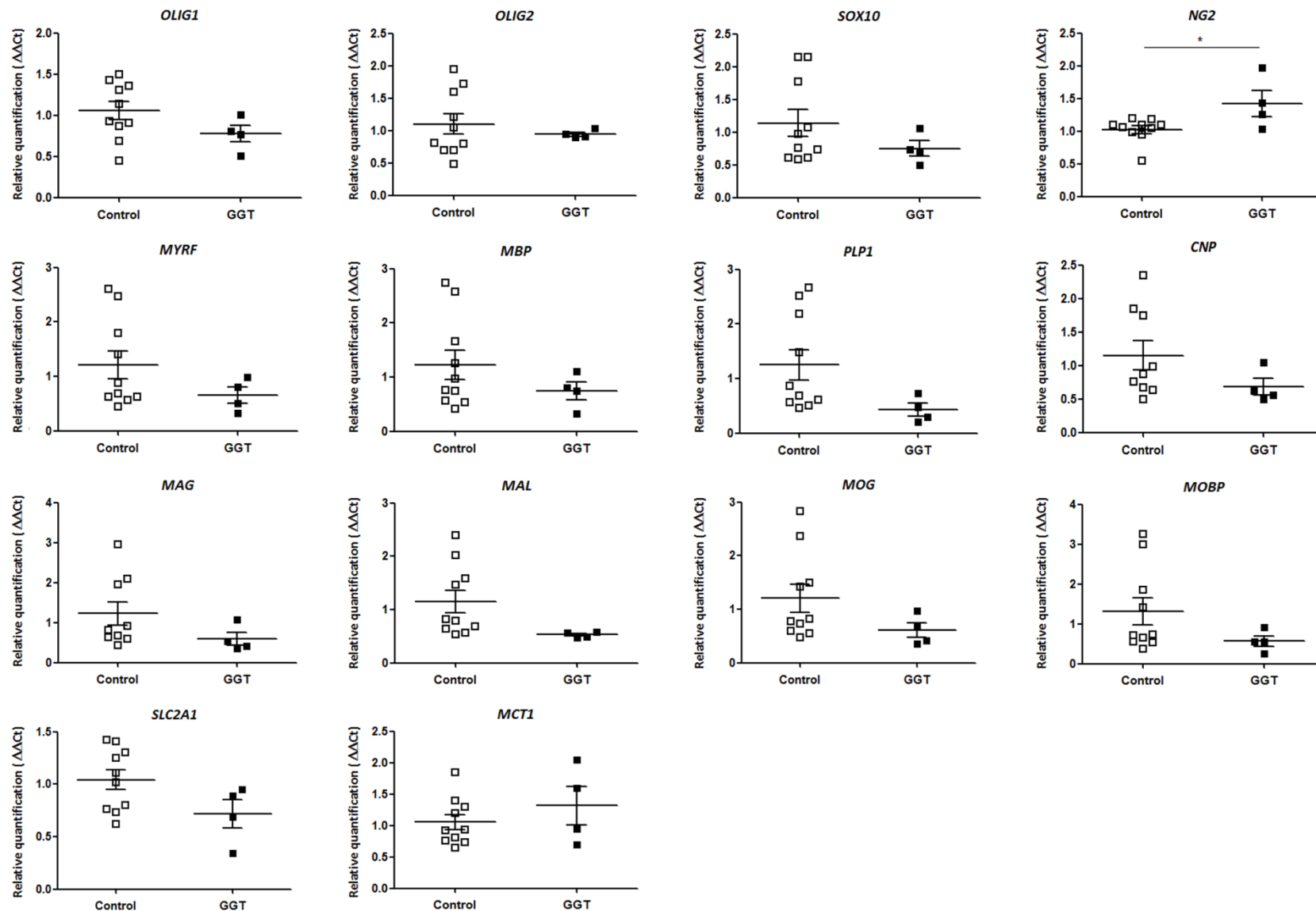

Supplement: Supplementary file 3 — Supplementary file3 Supplementary Figure 2 b) Dot graphs representing the expression of oligodendrocyte- and myelin-associated genes in frontal cortex area 8 in the four GGT cases linked to MAPT P301T mutation and 10 controls. NG2 mRNA expression is significantly increased in GGT cases; however, the majority of myelin-linked genes show a trend to decrease in disease cases. Student’s t test, p<0.05, ** p<0.01 and *** p<0.001 (PDF 314 kb) [file 401_2019_2122_MOESM3_ESM.pdf]

Supplementary Figure 3a

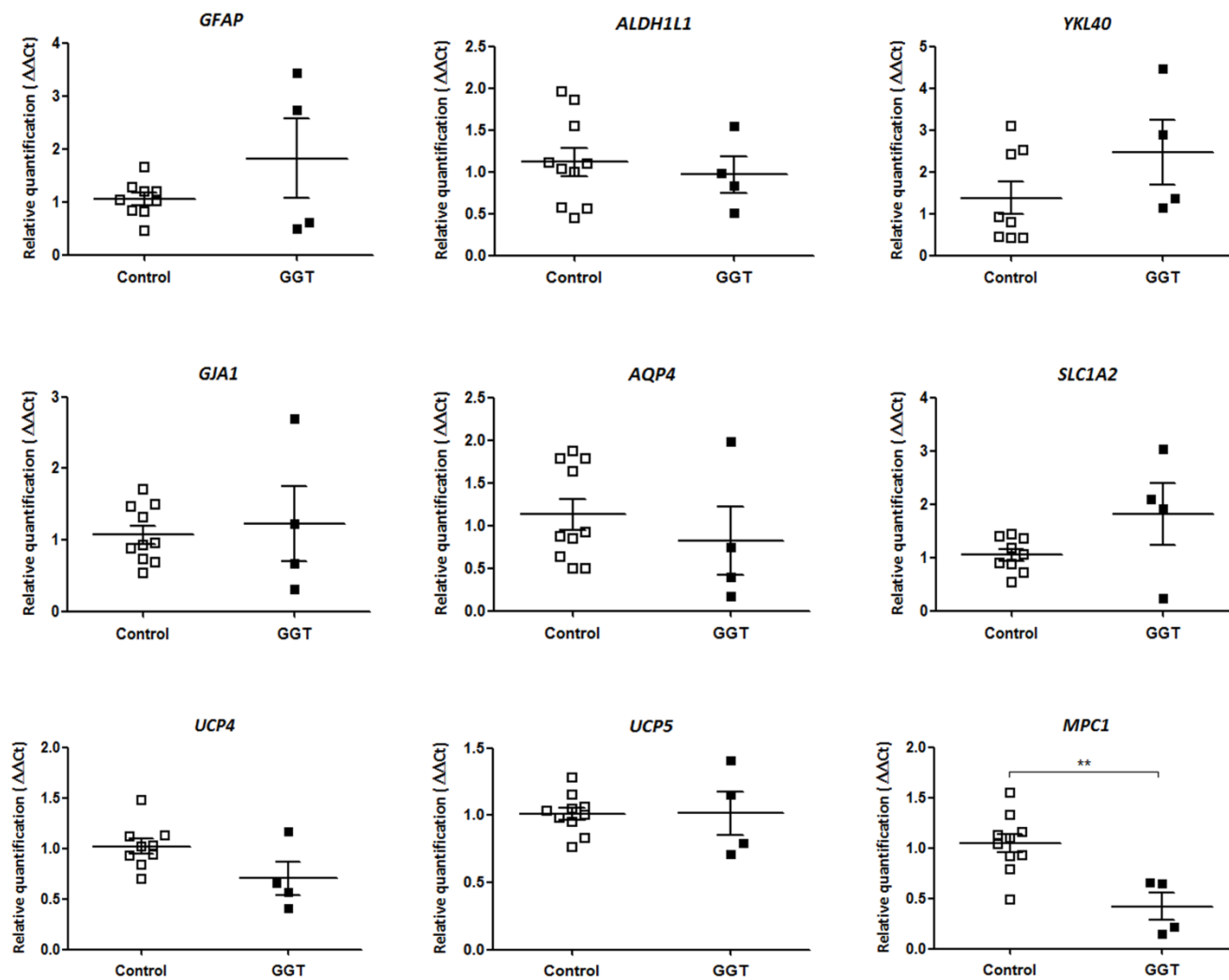

Supplement: Supplementary file 4 — Supplementary file4 Supplementary Figure 3 a) Dot graphs representing the expression of astrocyte-associated genes in the frontal subcortical white matter in the four GGT cases linked to MAPT P301T mutation and 10 controls. MPC1 mRNA is significantly decreased in GGT. Student’s t test, p<0.05, ** p<0.01 and *** p<0.001 (PDF 227 kb) [file 401_2019_2122_MOESM4_ESM.pdf]

Supplementary Figure 3b

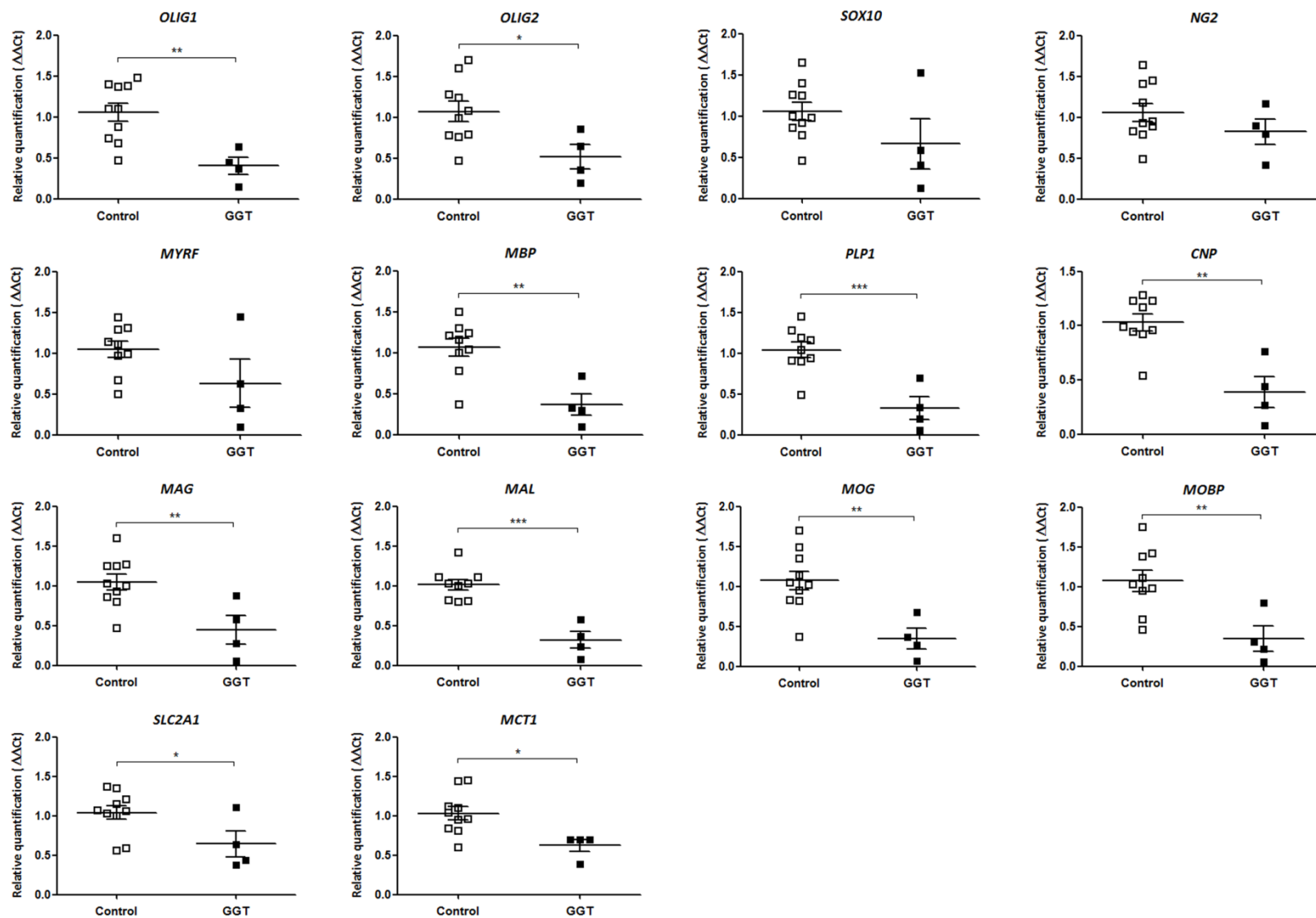

Supplement: Supplementary file 5 — Supplementary file5 Supplementary Figure 3 b) Dot graphs representing the expression of oligodendrocyte- and myelin-associated genes in the frontal subcortical white matter in the four GGT cases linked to MAPT P301T mutation and 10 controls shows significant reduction of OLIG1, OLIG2, MBP, PLP1, CNP, MAG, MAL, MOG, MOBP, SLC2A1, and MCT1 in GGT cases compared with controls. Student’s t test, p<0.05, ** p<0.01 and *** p<0.001 (PDF 317 kb) [file 401_2019_2122_MOESM5_ESM.pdf]

Supplementary Figure 4

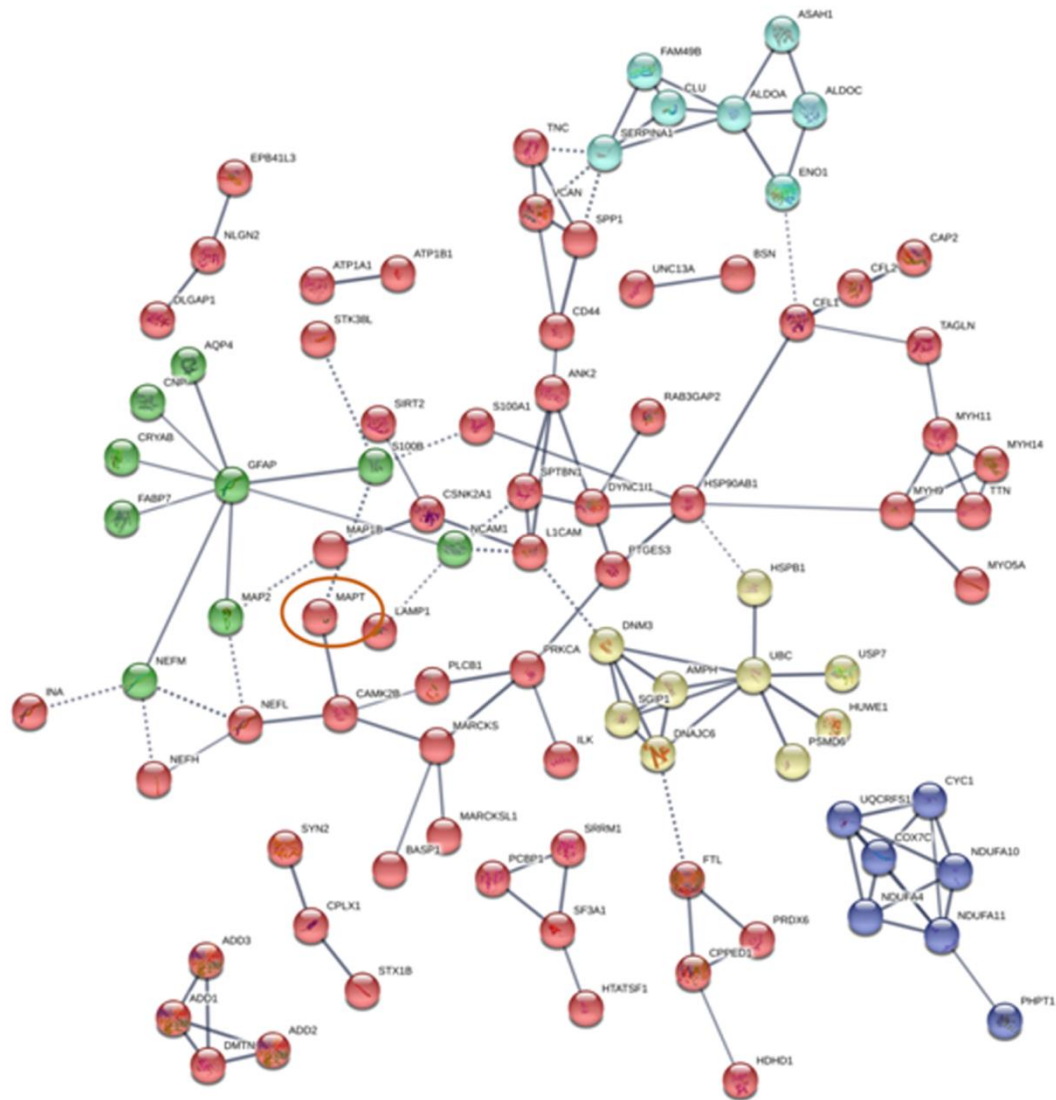

Supplement: Supplementary file 6 — Supplementary file6 Supplementary Figure 4 Protein interactome network for frontal cortex-modulated (phospho)proteome. Network analysis was performed submitting the corresponding protein IDs to the STRING (Search Tool for the Retrieval of Interacting Genes) software (v.10.5) (http://stringdb.org/). Proteins are represented with nodes and the interactions with continuous lines to represent direct interactions (physical), while indirect ones (functional) are presented by interrupted lines. All the edges were supported by at least one reference from the literature or from canonical information stored in the STRING database. To minimize false positives as well as false negatives, only interactions tagged as “high confidence” (> 0.7) in STRING database were considered. K means clustering was applied (PDF 111 kb) [file 401_2019_2122_MOESM6_ESM.pdf]

Supplementary Figure 5

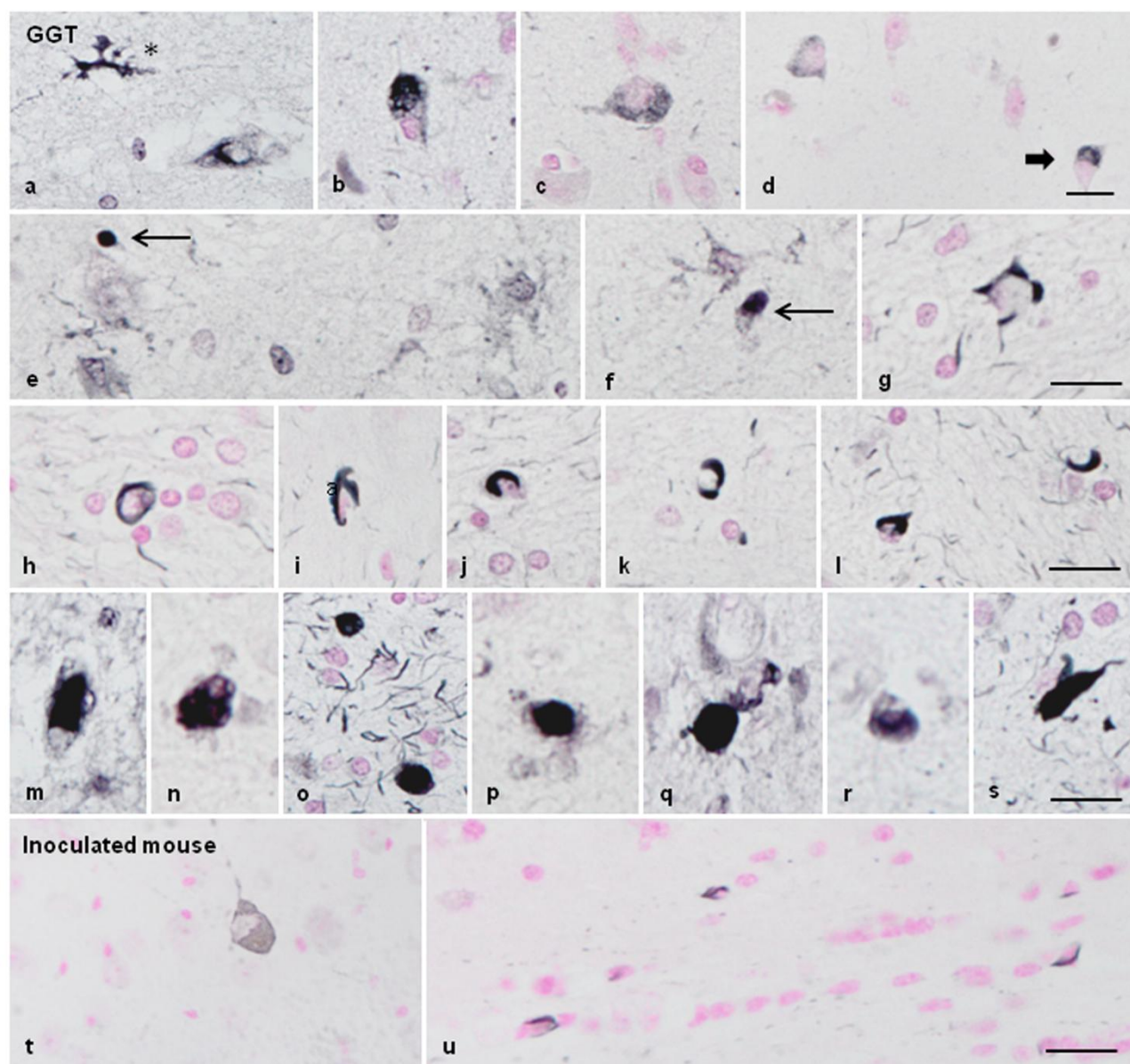

Supplement: Supplementary file 7 — Supplementary file7 Supplementary Figure 5 Gallyas staining of GGT cases (a-s) and inoculated mice with sarkosyl-insoluble fractions from GGT linked to MAPT P301T mutation (t, u). Neurons are variably stained in the cerebral cortex showing tangle-like morphology (a), dense granular staining (b and thick black arrow in d), weak and fine granular staining (c), faint difuse staining, or no staining (d). GAIs are negative, but a small subpopulation of astrocytes show faint granular Gallyas-positive staining in the distal region of astrocytic processes or in the cytoplasm of a few number astrocytes (e, f). Very rare astrocytes with perinuclear round Gallyas-positive deposits are seen in GGT linked to MAPT K317M mutation (g). Coiled bodies are positive (h-l), and there are GOIs (m-s) in every case. GOIs are also seen in e and f (thin black arrows). Gallyas-positive threads and bizarre oligodendroglial inclusions are also seen in GGT linked to MAPT K317M mutation (o, s). Gallyas-positive glial cells of unknown origin are rarely found in GGT linked to MAPT K317M mutation (asterisk in a). Mice inoculated with sarkosyl-insoluble fractions of GGT cases very rarely show faint positive neurons (t) and common Gallyas-positive coiled bodies (u). b, c, d e, h, i, m, n: GGT linked to MAPT P301T mutation, case 1; f, j, i, p: case 3; k: case 4; q, r: sGGT; g, l, o, s: GGT linked to MAPT K317M mutation. t, u: mouse inoculated at the age of 12 months with sarkosyl-insoluble fractions of GGT linked to MAPT linked to P301T mutation, case 1 (survival 6 months). Paraffin sections without counterstaining; a-d, bar = 25µm; e-l, bar = 10 µm; m-s, bar = 15 µm; t, s, bar = 20µm (PDF 180 kb) [file 401_2019_2122_MOESM7_ESM.pdf]

Supplementary Figure 6

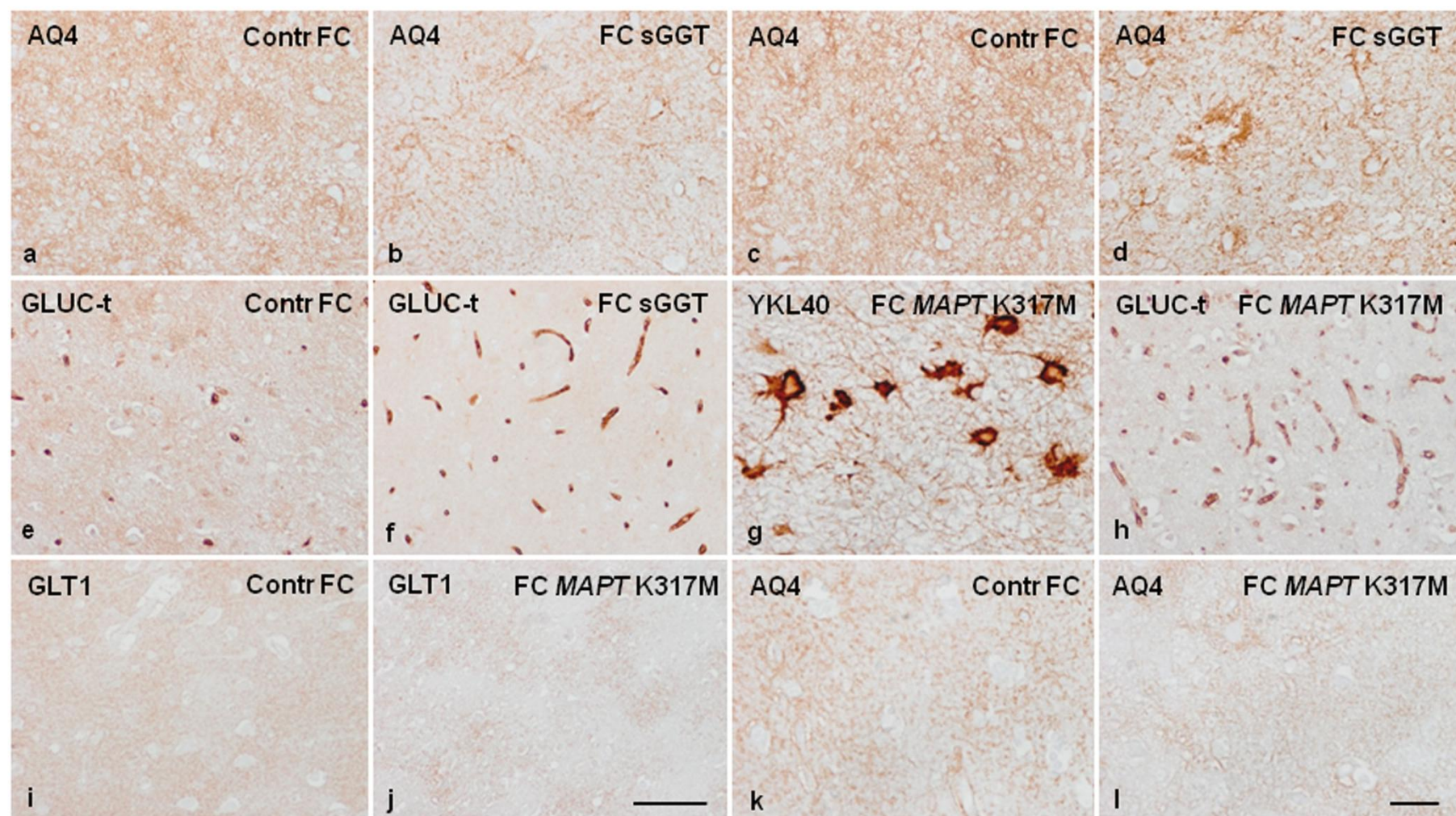

Supplement: Supplementary file 8 — Supplementary file8 Supplementary Figure 6 a-f: Aquaporin 4 (AQ4) and glucose transporter (GLUC-t) expression in the frontal cortex (FC) in sGGT and corresponding controls (Contr) processed in parallel. AQ4 immunoreactivity is reduced in sGGT when compared with controls, but AQ4 is still preserved around blood vessels (a-d). In control FC, GLUC-t is heavily expressed in capillaries and moderately so in the neuropil. GLUC-t is increased in sGGT because the higher number of capillaries when compared with controls, but GLUC-t immunoreactivity is decreased in the neuropil (e, f). g-l: YKL40, GLUC-t, glutamate transporter 1 (GLT1) and AQ4 in FC in GGT linked to MAPT K317M mutation and corresponding controls. YKL40-immunoreactive astrocytes show huge size and bizarre morphology (g) (compare Figure 3a for YKL40 immunoreactivity in the FC in controls). GLUC-t immunohistochemistry shows increased numbers of capillaries and decreased immunoreaction in the neuropil (h). GLT1 (i, j) and AQ4 (k, l) immunoreactivities are reduced in GGT linked to MAPT K317M compared with controls processed in parallel. Paraffin sections, slightly counterstained with haematoxylin; a, c, e, f, h, i and j, bar in j =70µm; b, d, g, k and l, bar in l = 35µm (PDF 306 kb) [file 401_2019_2122_MOESM8_ESM.pdf]

Supplementary Figure 7

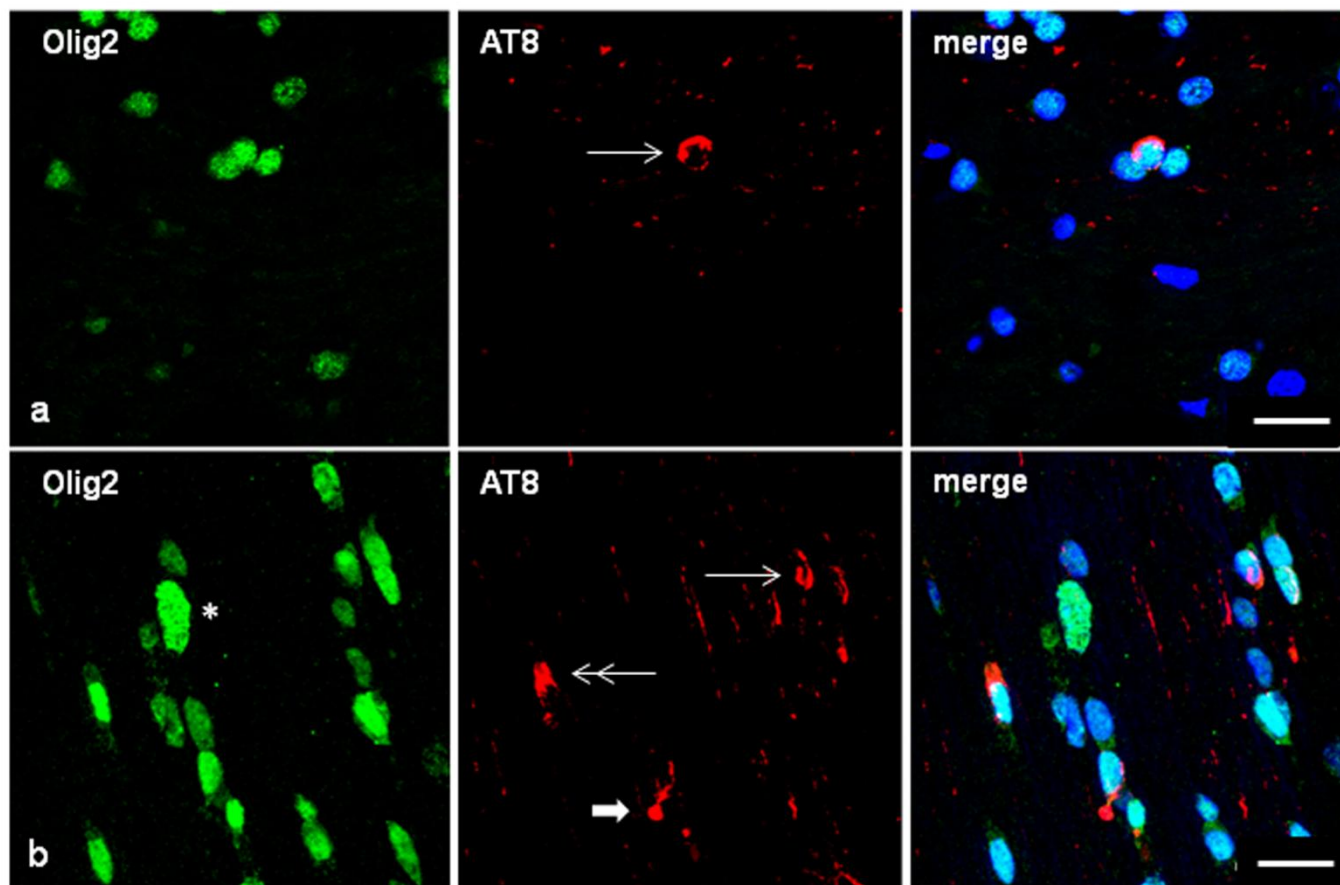

Supplement: Supplementary file 9 — Supplementary file9 Supplementary Figure 7 Double-labelling immunofluorescence and confocal microscopy to Olig2 (green) and AT8 (red) in the corpus callosum of mice inoculated with sarkosyl-insoluble fractions from GGT linked to MAPT K317M mutation shows typical coiled bodies (a and b)) (thin white arrows) and, more rarely, bizarre oligodendroglial tau-positive inclusions (thin white double-arrow) and tau-positive globular-like appendix in the cytoplasm (thick white arrow (b). The nucleus of one oligodendroglial cell is of a huge size (asterisk). Nuclei are counterstained with DRAQ5TM (blue). Paraffin sections, bar = 20 μm (PDF 117 kb) [file 401_2019_2122_MOESM9_ESM.pdf]
